# Hyperspectral Imaging Techniques for Lyophilization: Advances in Data‐Driven Modeling Strategies and Applications

**DOI:** 10.1002/advs.202508506

**Published:** 2025-07-23

**Authors:** Huiwen Yu, Prakitr Srisuma, Cedric Devos, Jie Wang, Allan S. Myerson, Richard D. Braatz

**Affiliations:** ^1^ Massachusetts Institute of Technology Cambridge MA 02139 USA

**Keywords:** analytic, biochemical engineering, data‐driven modeling, hyperspectral imaging, lyophilization

## Abstract

Lyophilization, aka freeze drying, is a key process used in the production of biotherapeutic products. The optimization of lyophilization formulations and operations is a slow process that could be accelerated by on‐line analytics. In recent years, hyperspectral imaging (HSI) has garnered increasing attention from both academia and industry in biopharmaceutical and food engineering fields. As a non‐invasive, rapid, non‐destructive, accurate, and automated tool that combines advantages from both spectroscopy and imaging techniques, HSI holds significant potential for analyzing and optimizing lyophilization processes and products. However, the huge and information‐rich datasets generated from HSI are difficult to be modeled and interpreted properly. This article reviews and discusses the literature on the application of HSI on lyophilization, and the strategies that use the resulting data to build models. Such strategies include preprocessing, spectral unmixing, classification and regression, and data fusion. From the data modeling and application perspectives, the current challenges and future prospects regarding HSI techniques for lyophilization are addressed. This article is intended to provide guidance and insights for non‐specialist researchers and engineers into leveraging HSI and the data‐driven modeling strategies for addressing a wide range of lyophilization‐related challenges.

## Graphical abstract

Lyophilization is a key process used in the production of biotherapeutic products. This article reviews and discusses the application of HSI on lyophilization, and the strategies that use the resulting data to build models. It is intended to provide guidance and insights for non‐specialist researchers and engineers into leveraging HSI and the data‐driven modeling strategies for addressing lyophilization‐related challenges.

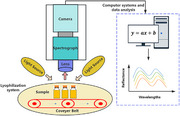

## 1 Introduction

Lyophilization, aka freeze drying, is an important process in the pharmaceutical and food industry for extending storage life and improving the stability of various products.^[^ ^1^, ^2^, ^3^ ^]^ Lyophilization primarily relies on the sublimation process for water removal, requiring the process to be carried out at lower temperatures compared to typical dehydration and drying techniques. As a result lyophilization is more effective at preserving the quality and structure of heat‐sensitive materials, such as pharmaceutical and food products. Lyophilization distinguishes itself from other common drying techniques (e.g., hot air, spray, or microwave drying) by preserving (to a large extent) the structure and composition of the product, thereby maintaining product quality and critical quality attributes.^[^ ^4^ ^]^ For example, the removal of water is important to ensure the long‐term stability of drug formulations.^[^ ^5^ ^]^ Similarly, in the food industry, water can cause spoilage and microbial growth and lyophilization is beneficial for storing items without the need for continuous refrigeration.^[^ ^6^ ^]^ By freezing the products and reducing the pressure to induce sublimation, lyophilization increases product stability and enhances the preservation for a wide range of products including biopharmaceuticals,^[^ ^7^, ^8^ ^]^ nutraceuticals, food,^[^ ^9^ ^]^ and biomaterials.^[^ ^10^ ^]^ Recently, advanced lyophilization techniques have been shown to enable long‐term and high‐temperature storage of lipid nanoparticles used in mRNA vaccine manufacturing.^[^ ^11^, ^12^ ^]^

Compared to traditional drying techniques, the lyophilization process is more complex and involves several distinct steps: freezing, primary drying, and secondary drying.^[^ ^13^ ^]^ During the freezing stage, most of the water in the liquid solution is converted to ice crystals. The duration of the freeze‐drying cycle, product stability, and crystallization of various components can be significantly affected during this stage. After the freezing stage, lyophilization proceeds to primary drying and concludes with secondary drying. During primary drying, ice is removed under vacuum through sublimation in a low‐temperature and low‐pressure environment. As the final step, secondary drying aims to remove unfrozen bound water under vacuum by desorption. Temperature, pressure, and moisture content are important variables during lyophilization that impact the quality of lyophilized products. Lyophilization is expensive, time‐consuming, and environmentally complex,^[^ ^8^, ^14^ ^]^ and the sterility requirements and sensitivity of processed products further impose practical difficulty in monitoring and characterization of lyophilization.^[^ ^15^ ^]^ Consequently, precise and efficient characterization, analytics, control, and monitoring of lyophilization‐related critical quality attributes remain challenging.

**Figure 1:**
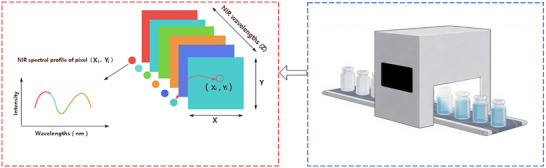
Hyperspectral image data structure with an example of NIR‐HSI for lyophilization process during pharmaceutical manufacturing. The conveyer belt is from BioRender.com.

As an emerging technique, hyperspectral imaging (HSI) has garnered increasing attention from both academia and industry in recent years. HSI, aka chemical imaging and spectroscopy imaging,^[^ ^16^ ^]^ combines the advantages of cutting‐edge spectroscopy and imaging techniques. Using a portion of the electromagnetic spectrum, HSI collects and processes image information from measured material in the manner of recording their continuous spectral profiles. Typically, HSI data are stored in a three‐dimensional format, with spatial dimensions denoted as *x* and *y*, and the spectral band dimension as *z*. This arrangement results in a 3D data cube structure. The typical HSI data structure with an example of Near‐Infrared (NIR)‐HSI for a lyophilization process during pharmaceutical manufacturing is illustrated in **Figure** **1**. Each HSI image represents information from a specific spectral band, and the HSI data cube is generated by combining a set of HSI images covering a wide range of wavebands. In the HSI image system, each pixel contains an individual spectral profile of the measured materials. These spectral profiles, aka spectral signatures, can be used for various purposes such as characterization, analytics, identification, control, and monitoring. Consequently, HSI generates a substantial amount of spectral image data. For example, an HSI dataset with a spatial resolution of 1000 by 1000 pixels contains 1 000 000 spectral profiles, each with a specific set of wavelength variables. Unlike traditional RGB imaging,^[^ ^17^ ^]^ which only captures information from red, green, and blue channels, HSI provides data across a wide range of wavebands, while also preserving spatial information for each pixel. Unlike multispectral imaging (MSI), HSI records abundant spectral information across a wide range of continuous wavebands, rather than targeting discrete and narrow wavebands. This advantage allows HSI to capture more continuous, detailed, and accurate spectral characteristics of materials compared to MSI techniques.^[^ ^18^ ^]^ Additionally, HSI techniques have had significant benefits from its high spectral resolution and high temporal resolution,^[^ ^19^ ^]^ making it a powerful tool for a wide range of types of practical analytics and process control tasks.

**Figure 2:**
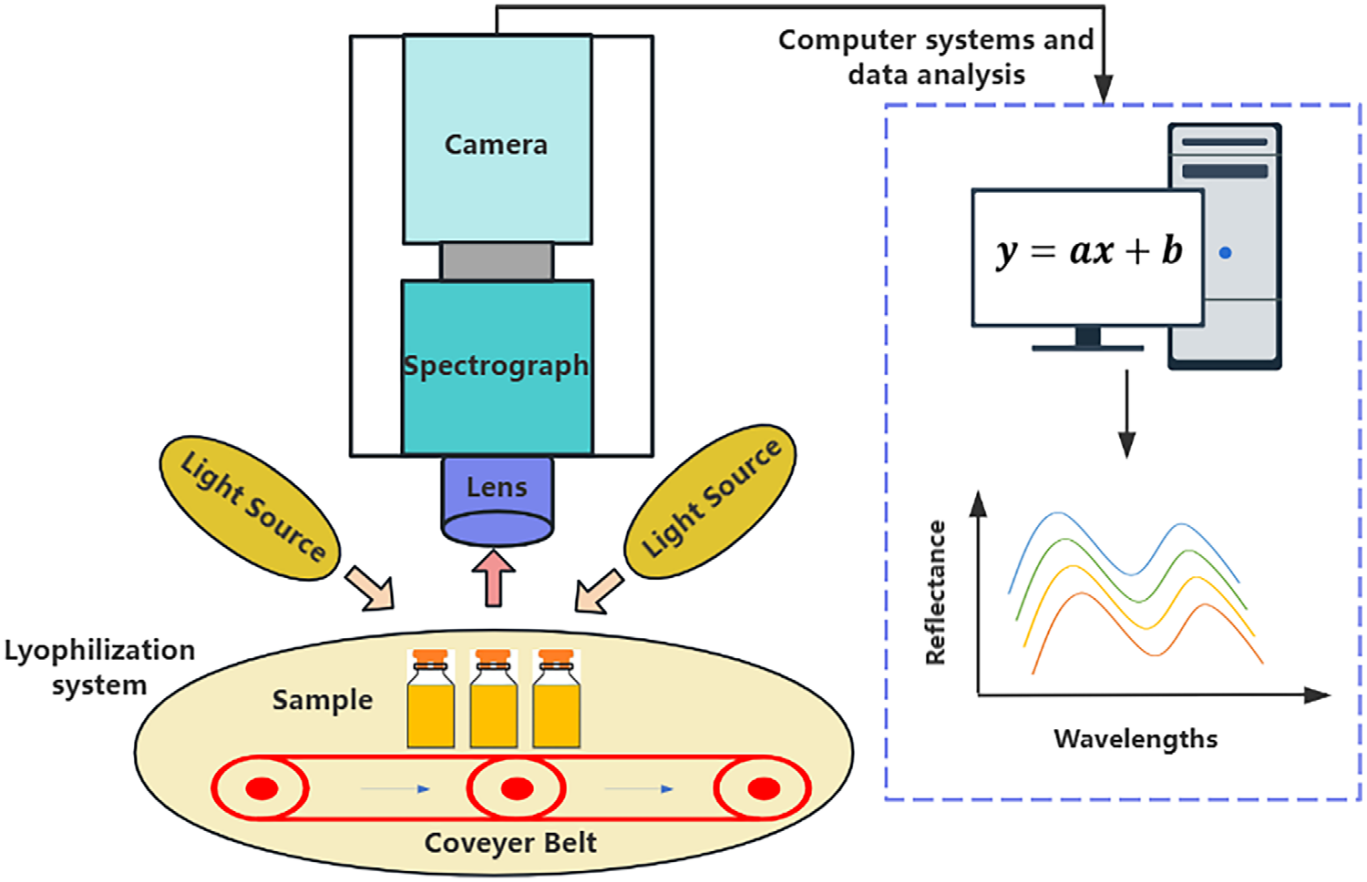
The schematic illustration of a typical HSI system for lyophilization applications. The “*y* = *ax* + *b*” denotes that data‐driven models for most HSI applications are linear.

The successful early application of HSI dates back to the 1980s in the field of remote sensing.^[^ ^20^ ^]^ Over the past two decades, HSI has found widespread use in characterizing, identifying, detecting, and monitoring a diverse range of complex biological materials and products.^[^ ^21^, ^22^, ^23^ ^]^ A typical HSI system comprises a light source, an imaging spectrograph system, and a camera system.^[^ ^24^ ^]^ In lyophilization applications, samples are illuminated by the light source. The front lens of the imaging spectrograph system then image the samples into the transmission spectrograph. The produced spectrum is captured by the camera system, which includes an array detector or other sensors. Continuous measurements over time generate the complete HSI data for the samples, known as the “hypercube.” **Figure** **2** provides a schematic illustration of an HSI system for lyophilization applications. HSI systems can be classified based on various criteria, such as the types of detectors, spectral range, measurement modes, and image acquisition modes. For example, there are whisk broom scanners, push broom scanners, snapshot HSI, and filter‐based HSI, each differing in image acquisition mode.^[^ ^25^ ^]^ In push broom scanners, the camera acquires images in a linear scanning manner, moving over the samples. Whisk broom scanners scan the full spectrum pixel by pixel, while filter‐based HSI performs a staring scan of the scene.^[^ ^26^ ^]^ Filter‐based HSI relies on optical band‐pass filters and primarily operates on stationary platforms, whereas the first two types of broom scanners are suitable for moving environments, such as conveyor belts. Snapshot HSI, on the other hand, does not involve scanning and captures the HSI image in a single snapshot, characterized by fast acquisition time and easy implementation. Many types of HSI have been developed over the years, including Ultraviolet (UV)‐HSI, Visible (Vis)‐HSI, NIR‐HSI, Mid‐infrared (MIR)‐HSI, Raman‐HSI, Fluorescence‐HSI, and Fourier transform infrared (FT‐IR)‐HSI. These HSI systems vary in spectral range or measurement mode and have been used for characterization, analysis, monitoring, and control purposes in various biochemical applications.^[^ ^27^, ^28^ ^]^

**Table 1:** Summary of HSI for the lyophilization of pharmaceutical compounds.

Study	Sample	Application	Spectral type	Wavelength	Modeling strategies
^[^ ^36^ ^]^	Mannitol, sucrose, lysozyme, bovine serum albumin	Determine water content, study crystallization of sucrose	NIR	970–2500 nm	PCA and PLS
^[^ ^2^ ^]^	Mannitol and sucrose	Determine water content, explore the distribution of β‐mannitol and δ‐mannitol	NIR	900–1700 nm	PCA and PLS
^[^ ^120^ ^]^	Mannitol	Polymorphic forms	Raman	514 nm	PCA and LCE
^[^ ^121^ ^]^	Furazolidone	Characterize the formation of complexes with cyclodextrins	Raman	532 nm (N/A)	N/A
^[^ ^22^ ^]^	Pantothenate kinase	Investigate enzyme immobilization	Raman	532/785 nm (N/A)	PCA and PLS
^[^ ^118^ ^]^	Iburofen	Study distributions and transformations of iburofen and mannitol	Raman	785 nm (N/A)	PCA and PLS
^[^ ^31^ ^]^	Human serum albumin	Detect particulate matters	X‐ray	N/A	N/A
^[^ ^131^ ^]^	Pantothenate kinase	Investigate enzyme immobilization	Raman	532/785 nm	NMF
^[^ ^5^ ^]^	Dextran, sucrose, polyvinylpyrrolidone, D‐mannitol and lysozyme	Phase separation	Raman	785 nm	SNV
^[^ ^127^ ^]^	Lysozyme and trehalose	Homogeneity	NIR	1200–2400 nm	Savitsky‐Golay filter, PCA and PLS
^[^ ^126^, ^128^ ^]^	Polyvinylpyrrolidone, dextran, Ficoll, bovine serum albumin, trehalose and lysozyme	Phase separation	Raman	785 nm	SNV
^[^ ^130^ ^]^	Lysozyme, heavy water, trehalose, and glycerol	Protein stabilization	Raman	514.5 nm	Baseline correction, polynomial fitting
^[^ ^129^ ^]^	Lysozyme, heavy water, trehalose, and sucrose	Protein stabilization	Raman	514.5 nm	Intensity integration

**Table 2:** Summary of HSI for lyophilization applications in food industries and other biochemical related fields.

Study	Sample	Application	Spectral type	Wavelength	Modeling strategies
^[^ ^32^ ^]^	Broccoli florets	Quantification and localization of bioactive compounds in florets	Vis‐NIR, NIR	950–1650 nm	SNV, PCA, PLS
^[^ ^137^ ^]^	Shiitake mushrooms	Quantitative prediction of water fractions during processing	UV, NIR	405–970 nm	PLS, back propagation neural network, LS‐SVM
^[^ ^142^ ^]^	*Brassica juncea* leaves	Quantification and localization of five representative functional components	Vis‐NIR	400–1000 nm	Savitzky‐Golay filter, SNV, MSC, 1 derivative, 2 derivative, normalization, and PLS
^[^ ^141^ ^]^	*Ctenopharyngodon idella* fillets	Textural feature prediction	Vis‐NIR	400–1000 nm	PLS
^[^ ^6^ ^]^	*Ctenopharyngodon idella* fillets	Determination of moisture content under different freeze drying periods	Vis‐NIR	400‐1000 nm	MSC, SNV, PLS using leave‐one‐out cross validation
^[^ ^140^ ^]^	Apple slices	Analysis of heterogeneity of apple slices (dry content matter, and functional components)	NIR	1000–2500 nm	PCA and LOO‐PLS
^[^ ^144^ ^]^	Halloumi cheese	Species identification for authenification	Vis‐NIR	400–1000 nm	PCA and hierarchical cluster analysis
^[^ ^145^ ^]^	Black tea leaves	Quantification and localization of chemical and physical components	Vis‐NIR	400–1000 nm	MSC, 2 derivative, min‐max normalization, Monte Carlo based non formative variable elimination, shuffled frog leaping algorithm, PLS, SVR, and RF.
^[^ ^143^ ^]^	*Agaricus bisporus*	Quantification and localization of moisture content throughout process	Vis‐NIR	400–1000 nm	SVM, SCARS, and MSC
^[^ ^148^ ^]^	Chili pepper	Determination of moisture content	NIR	874–1734 nm	PLS, ELM, and LS‐SVM
^[^ ^152^ ^]^	Lipids in kidney tissues	Spatial lipidomics analysis	MIR	2500–25,000 nm	K‐means clustering, PCA, lasso regression, 2 derivative, Savitzky‐Golay filter
^[^ ^153^ ^]^	Varicella‐zoster viruses	Virus fingerprinting and compositional analysis	MIR	2500–25,000 nm	N/A

HSI is a non‐invasive, rapid, non‐destructive, highly accurate, and automated tool suitable for precise characterization, control, and monitoring in lyophilization. Unlike traditional chemical analysis, HSI implementations do not necessarily require complex sample preparation processes and chemical labeling.^[^ ^29^, ^30^ ^]^ As a result, HSI can significantly reduce labor costs and be economically cost‐effective. Currently, the HSI systems used in lyophilization are mainly from visible light to near‐infrared bands. The spectral resolution of these HSI systems covers from nanometers to centimeters representing different detection capabilities and different detection times. Studies have demonstrated the effectiveness of HSI in capturing meticulous chemical and physical information during and after lyophilization. Particulates as small as 2 mm have been detected by current HSI systems.^[^ ^31^ ^]^ Root mean squared prediction error as low as 0.15 has been reported for HSI.^[^ ^32^ ^]^ Highly sensitive HSI detection of physical and chemical information is crucial for lyophilization applications. In pharmaceutical production, drugs are highly sensitive to moisture and prone to hydrolysis and degradation and regulatory agencies impose strict limits on residual moisture levels.^[^ ^33^ ^]^ Precise HSI detection of residual moisture during the lyophilization process is essential for ensuring drug stability, efficacy, and safety throughout their shelf life. Additionally, trace foreign matter such as fibers can easily contaminate lyophilization drugs, posing a risk to drug safety. Highly sensitive HSI enhances the detection of such ultra‐low variation of contaminants, reducing the risk of contamination in pharmaceutical production and improving overall product quality. HSI has been demonstrated for water to ice conversion, product crystallization, solid‐state characterization, moisture content determination, protein unfolding, and investigation of interacting phenomena.^[^ ^2^, ^34^, ^35^, ^36^, ^37^ ^]^ Leveraging its extended wavelength range and spatial imaging advantage, HSI reveals material properties that are not apparent through other imaging techniques, making it a valuable tool in the lyophilization analysis for pharmaceutical systems. HSI enables analysis of complex heterogeneous samples often encountered in lyophilization.^[^ ^38^ ^]^ Furthermore, HSI is well‐suited for in‐line monitoring of lyophilization from start to finish, enabling simultaneous monitoring of multiple various factors or parameters, thus facilitating automated inspection. Compared to conventional analysis techniques, HSI presents distinct advantages such as precision, speed, and non‐destructive analysis.^[^ ^39^ ^]^ Additionally, HSI can generate comprehensive visualization maps of the product that facilitate detailed qualitative and quantitative assessments of the homogeneity of critical quality attributes, whereas traditional spectral techniques provide information limited to specific detection areas.^[^ ^2^ ^]^ This feature is specifically important for lyophilization in which non‐uniformity exists throughout the process, e.g., thermal gradients resulting from vials heated or cooled by the bottom shelf. Consequently, HSI holds the potential to induce a paradigm shift from a quality‐by‐testing framework to a quality‐by‐design methodology, a transition particularly advocated for in the pharmaceutical industry.^[^ ^2^ ^]^

**Figure 3:**
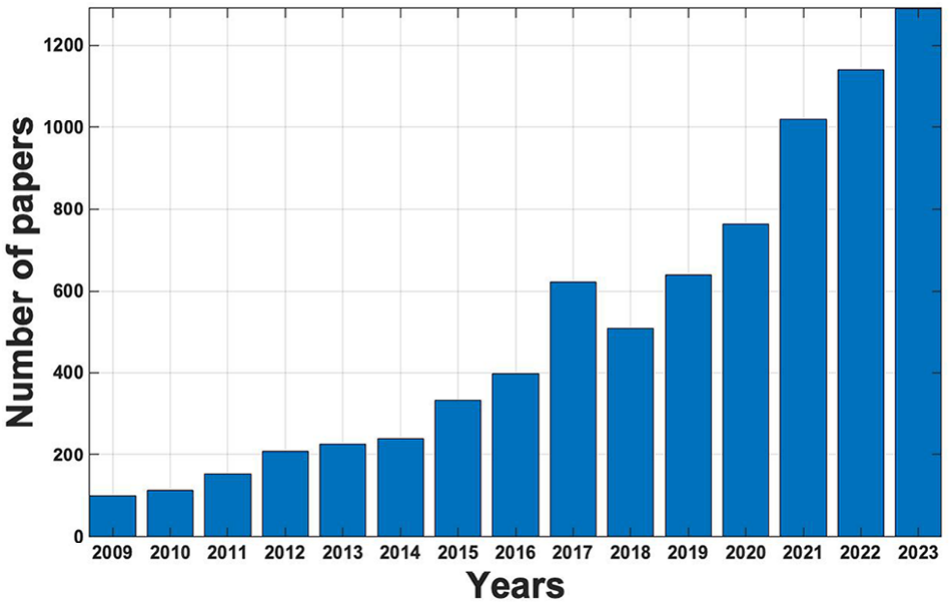
The number of papers indexed in Google Scholar on the subjects of “hyperspectral imaging” and “freeze drying” in the past 15 years.

A literature search via Google Scholar using the key words “hyperspectral imaging” and “freeze drying” for the period from 2009–2023 illustrates a significant increase in the literature over the past 15 years (**Figure** **3**), indicating growing interest in HSI techniques within the lyophilization community. The number of publications continues to show an upward trend, indicating that interest in this area is high enough to justify a review of progress and directions for continued efforts.

This article does not aim to comprehensively cover all topics related to HSI data modeling techniques, nor does it provide an exhaustive review of HSI technique itself. Instead, we offer a perspective on typical HSI data modeling strategies that have the potential to address lyophilization‐related HSI modeling challenges. Additionally, we emphasize the value of direct applying HSI techniques in lyophilization contexts. The paper is organized as follows. In the second section, we provide explanations for the notation and mathematical symbols used. Moving on to the third section, we offer an overview and discussion of data‐driven modeling strategies for HSI data analysis with the application potential for lyophilization contexts. This section covers various modeling strategies, including HSI data preprocessing methods, spectral unmixing modeling, classification and regression methods, and data fusion strategies. The fourth section summarizes and discusses recent HSI applications in lyophilization to pharmaceuticals, food, and other biological systems. Following this, we delve into current challenges and future perspectives related to HSI data modeling and applications in lyophilization. Finally, we conclude the paper.

## 2 Notations and Symbols

This article adheres to conventional notation and nomenclature.^[^ ^40^, ^41^ ^]^ Scalars are denoted by italic non‐bold letters e.g., *X*, and vectors are represented using bold lowercase letters, e.g., **x**. Matrices are denoted by bold uppercase letters such as **X**, with elements denoted as *X*_*ij*_. Tensors are denoted by bold capital letters with an underline, e.g., $\underline{X}$. The mathematical symbols ○, ⊗, and ⊙ correspond to the outer, Kronecker, and Khatri‐Rao products, respectively. The Kronecker product is also referred to as the matrix direct product and the Khatri‐Rao product is column‐wise Kronecker product.^[^ ^42^ ^]^ For matrices **A** and **B** with the dimensions of *I* by *F* and *J* by *F*, their Khatri‐Rao product of **A** and **B** is defined by

(1) $$\begin{array}{ccc} \mathrm{KR}(AB) & = & A⊙B=[\mathrm{vec}\left(a_{1}b_{1}^{⊤}\right)\;\cdots \;\mathrm{vec}\left(a_{F}b_{F}^{⊤}\right)]\\ & & =[\left(a_{1}⊗b_{1}\right)\;\cdots \;\left(a_{F}⊗b_{F}\right)] \end{array}$$

where **a**_*F*_ and **b**_*F*_ are the *F*th column vector from matrices **A** and **B**, ^vec^ is the vectorization of matrices, and ⊤ denotes the transpose. The Kronecker product of **A** and **B** is defined by

(2) $$\mathrm{Kron}\left(AB\right)=A⊗B=\left[\begin{array}{c} a_{11}B\;\cdots \;a_{1F}B\\ \vdots \;\ddots \;\vdots \\ a_{I1}B\;\cdots \;a_{IF}B \end{array}\right].$$

## 3 Data‐Driven Modeling Strategies

### 3.1 HSI Data Pre‐Processing

The quality of HSI image data is influenced by various factors, including the lyophilization products or process, light source, product interface, sensor equipment, and any artifacts introduced by humans. These combined impact factors manifest as a set of analytical and modeling challenges in practical lyophilization HSI data analysis, including scattering effects, spectral noise, abnormal signals (e.g., spike signal and dead pixels), image compression, and image distortion. Preprocessing plays a crucial role in lyophilization HSI data modeling and analysis. Whether the goal is classification, regression, or exploratory analysis, proper preprocessing consistently improves model performance, reduces model fitting error, and enhances understanding of the measured lyophilized products or processes.

Due to light deviating from a straight trajectory into different paths, unexpected and undesired variations can occur in the spectral data, leading to nonlinearity scattering effects.^[^ ^43^ ^]^ Over the years, several scattering correction methods have been developed to address this issue in HSI data analysis. The commonly used methods are standard normal variate (SNV) and multiplicative scatter correction (MSC), which focus on solving addictive and multiplicative scattering, respectively.^[^ ^44^ ^]^ The SNV method is a normalization method that subtracts each spectrum by the mean spectrum and divides by the standard deviation. However, SNV cannot handle multiplicative scattering effects. To address this limitation, the MSC method corrects the spectra affected by multiplicative scattering, by fitting a linear model between the individual spectra and a reference spectrum. Specifically, the model is

(3) $$x_{\mathrm{cor}}=\frac{x_{\mathrm{ind}}-b_{0}}{b}=x_{\mathrm{ref}}+\frac{1}{b_{\mathrm{ref}}}e$$

where *x*_cor_ is the corrected spectrum value in the specific wavelength, *x*_ind_ is an individual spectrum value in the specific wavelength, *x*_ref_ is the reference spectrum value in the specific wavelength, *b* and *b*_0_ are the estimated correction coefficients, and *e* is the modeled error of the individual spectrum. In recent years, some variants/extended versions of MSC and SNV have been developed, including dynamic localized SNV, partial peak SNV, SNV with detrending, extended MSC, and weighted MSC.^[^ ^45^, ^46^, ^47^ ^]^ These methods can increase the performance and applicability of MSC and SNV normalization methods for lyophilization HSI image data preprocessing. In addition to these methods, derivative‐based approaches can also address scattering effects. The first derivative and second derivative methods are popular due to their ability to remove both additive and multiplicative effects.

Spectral noise can be a challenge in HSI data preprocessing. Derivative methods, such as the Savitzky–Golay method,^[^ ^48^ ^]^ are employed for denoising in HSI data analysis. The Savitzky–Golay method selects a window around a data point and calculate its projection onto a polynomial fit of the window points. This step allows estimation of derivatives of any order for the data points. As a smoothing technique, the Savitzky–Golay method effectively reduces spectral noise level. However, the choice of local window size and the orders of derivatives significantly impacts the performance of the Savitzky–Golay method and its variants. In addition to the Savitzky–Golay method, recent studies have demonstrated improved performance of smoothing and denoising methods in reducing noise in HSI data analysis. These include wavelet transform (WT), denoising autoencoder (DAE), empirical mode decomposition (EMD), and deep learning methods.^[^ ^49^, ^50^ ^]^

Abnormal signal, e.g., spike, characterized by a sudden rise to a large magnitude followed by a dramatic fall, is a challenging task that needs to be handled in HSI data preprocessing. The spikes can occur due to various factors, including abnormal behaviors of the specific HSI detector and environmental conditions of the lyophilization measurement. To address spikes in Raman HSI, Goedhart et al.^[^ ^51^ ^]^ proposed a method for the identification and removal of cosmic ray spikes. This method combines identification and removal steps using a low‐pass filter, Gaussian masked kernel regression, and third‐order P‐spline on the signals. Lopez et al.^[^ ^52^ ^]^ developed an automated method based on peaks widths and prominences. They set a threshold value for these features or use the ratio of these two features as the threshold value to detect the spikes in spectral signal analysis.The new spikes handling method shows competitive performance in real HSI data analysis. Another common abnormal signal issue is the so‐called dead pixel. It is caused by abnormalities in the specific detector. The occurrence of dead pixels varies among different types of detectors. For example, it is reported that NIR detectors tield approximately one percentage dead pixels during HSI measurements.^[^ ^53^ ^]^ Recently, double low rank matrix decomposition methods^[^ ^54^ ^]^ have been applied for handling dead pixels. In these methods, general matrix decomposition models are used, and dead pixels are treated as sparse components. A simple and intuitive way to handle spikes or dead pixels is to use interpolation methods. Specifically, the median or mean value of the subwindow points or the neighbor pixels is used for interpolating these abnormal signals. Moreover, it is not unusual to encounter the image distortion in collected lyophilization HSI image dataset. The reasons for distortion can be diverse. HSI equipment placed in a moving lyophilization process, such as an HSI camera on conveyor belts in pharmaceutical manufacturing, likely produces the distorted images. Additionally, the use of complex optics such as a co‐focal camera probably also results in HSI images with distortion.^[^ ^53^ ^]^ The typical approach to handle HSI image distortion involves identifying reference points in the images and implementing interpolation using conventional methods such as bicubic interpolation.^[^ ^55^ ^]^ The combination of reference correction and interpolation techniques is widely used for addressing HSI image distortion. However, special attention should be given to the selection of a reference, which significantly affects the method performance.

Image compression plays an important role in lyophilization HSI data preprocessing. As mentioned in the Introduction section, the HSI images often arrive in the hands of analysts or engineers as large amounts of data. Dealing with big data presents challenges related to data storage, data transfer, and computational costs. HSI image compression techniques are preprocessing methods specifically designed to address the compression challenge during the preprocessing stage while minimizing loss of relevant and important HSI information. Wavelet transform is a mature technique for lyophilization HSI image compression, leveraging its advantageous filter properties to reduce redundant information. Both continuous and discrete wavelet transform methods have been applied to compress HSI images. For example, support vector regression has been combined with the 3D wavelet transform for large‐scale HSI image compression.^[^ ^56^ ^]^ This wavelet transform‐based approach preserved spatial and spectral information while removing redundancies from the HSI image data. Variable selection methods also contribute to lyophilization HSI image compression. Techniques commonly used in statistics and chemometrics – such as genetic algorithm, interval partial least squares(IPLS), recursive least squares, adaptive selection methods – have demonstrated competitive performance in this context.^[^ ^57^ ^]^ Another approach involves dimensional reduction techniques, which transform the high‐dimensional HSI image into lower dimensional spaces. Principal component analysis (PCA) is a typical method for this purpose.^[^ ^58^ ^]^ PCA decomposes lyophilization HSI image data into a set of principal components, comprising a score matrix and a loading matrix. The principal components capture the essential compressed physical/chemical information from lyophilization products or process, while the residuals represent the redundant image details. In practical lyophilization HSI data preprocessing, the choice of HSI image compression methods depends on the types of HSI data, analytic and modeling requirements, and noise level. The ways of determining the size of important variables impact the performance of variable selection methods. With this regard, prior knowledge of the data from the specific lyophilization products or process may be necessary. Moreover, in dimensional reduction methods such as PCA, determining the appropriate number of components is not always intuitive. A combination of statistical diagnostic (e.g., scree test) and prior knowledge on the data helps make informed decisions. It is worth noting that the individual preprocessing technique can address various HSI image problems based on specific modeling needs. For example, while the wavelet transform method is commonly used for image compression, it also serves as a spectral denoiser in HSI data analysis. In real‐world scenarios, different preprocessing techniques often coexist within the same lyophilization HSI modeling task, addressing issues such as scattering effects, abnormal signals, and data redundancy simultaneously.

In recent years, high‐order tensor‐based methods have shown potential for HSI image preprocessing. For example, tensor ring decomposition provides lyophilization HSI image compression. Essentially, the tensor ring model aims to capture a sequence of latent tensors, referred to as *core tensor arrays* $\underline{Z}=\left[{\underline{Z}}^{1},{\underline{Z}}^{2},{\underline{Z}}^{3},\cdots ,{\underline{Z}}^{n}\right]$, from the high‐order tensor data, where ${\underline{Z}}^{k}\in {ℜ}^{r_{k}\times I_{k}\times r_{k+1}}$, *r* = [*r*_1_, *r*_2_, *r*_3_, …, *r*_*n*_] denotes the tensor ring model rank, and *I*_*k*_ represents each dimension of the high‐order tensor. For a third‐order tensor $\underline{X}$, the tensor ring model can be expressed element‐wise by

(4) $${\underline{X}}_{\left(i1,i2,i3\right)}=\mathrm{Tr}\left(Z_{i1}^{1}Z_{i2}^{2}Z_{i3}^{3}\right)=\mathrm{Tr}\;\left(\prod_{k=1}^{n}Z_{ik}^{k}\right)$$

where Tr(⋅⋅⋅) is the trace operation for matrices, ${\underline{X}}_{\left(i1,i2,i3\right)}$ is the element located at position (*i*1, *i*2, *i*3) in tensor $\underline{X}$, and $Z_{ik}^{k}$ corresponds to the *ik*th lateral slice matrix of the interested tensor array ${\underline{Z}}^{k}$. In recent work, tensor ring decomposition was used to approximate and constrain the grouped non‐local tensor, which is an additional term in the HSI compression function. This term has both spatial‐spectral and non‐local dimension correlations in HSI image compression. The tensor ring model‐based HSI image compression method preserves the initial tensor structure of HSI image data, enhances basis learning of materials constituents in the image data, and achieves the precise HSI image compression reconstruction.^[^ ^59^ ^]^ Beyond HSI image compression, high‐order tensor methods have also been explored for HSI denoising. The rank‐(*L*_*r*_, *L*_*r*_, 1) block term tensor decomposition is a powerful model for lyophilization HSI denoising. The HSI tensor is decomposed as a linear combination of the so‐called low multilinear rank terms. The rank‐(*L*_*r*_, *L*_*r*_, 1) block term tensor decomposition model can be expressed as

(5) $$\underline{X}=\sum_{f=1}^{F}A_{f}B_{f}^{⊤}\circ c_{f}$$

where $\underline{X}\in {ℜ}^{I\times J\times K}$, *F* is the rank of block term tensor decomposition model, **A**_*f*_ is a *I* by *L*_*r*_ matrix, **B**_*f*_ is a *J* by *L*_*r*_ matrix, and **c**_*f*_ is a *K* by 1 vector. This model has been extended to form the nonlinear transformed block term tensor method for handling mixed HSI noise.^[^ ^60^ ^]^ The HSI noise minimization function is revised by introducing a regularized term and a gradient‐map tensor term. The new noise minimization function is constrained by an element‐wise nonlinear transform block term tensor 6 decomposition approximation.

In the past few years, other tensor models have been explored to address different HSI preprocessing challenges.^[^ ^61^, ^62^ ^]^ The emerging of high‐order chemometrics and mathematical methods provides new modeling tools for tackling complex lyophilization HSI image preprocessing tasks.

### 3.2 HSI Unmixing Modeling

The initial information for gaining insights from HSI analysis regarding a specific lyophilization product or process lies within the image itself. However, raw hyperspectral image does not directly reveal microscopic components, such as information on pure spectral signatures and abundances. These details are of high interest to lyophilization users in practice. Hyperspectral unmixing refers to a set of techniques aimed at extracting detailed information on spectral signatures from the lyophilization image data. Since spectral signatures in lyophilization hyperspectral images typically result from a mixture of different substances, hyperspectral unmixing seeks to identify the pure spectrum of each substance, often referred to as “endmembers”, associated with each pixel. Additionally, the fractional abundance of each endmember is estimated.^[^ ^63^ ^]^ Linear unmixing methods serve as widely used tools for unmixing the hyperspectral image. One of the most popular and straightforward linear unmixing approaches is the linear mixture model (LMM).^[^ ^64^ ^]^ In the LMM, each pixel in the image is assumed to be a linear combination of the endmembers scaled by their respective abundances. The interactions between different endmembers are considered negligible. Mathematically, the LMM model can be expressed in matrix form as

(6) X=AB+E

where **X** is a *S* by *Q* HSI matrix, **A** is a *S* by *V* endmember matrix or pure spectral signature matrix, **B** is a *V* by *Q* abundance matrix, and **E** is a *S* by *Q* error matrix with the same dimension as **X**. Due to the mathematical simplicity and intuitive model structure, LMM models are widely used to perform the linear lyophilization HSI data unmixing. Over the years, some LMM‐based or LMM‐like methods have been developed and applied to HSI data modeling, including Vertex Component Analysis (VCA),^[^ ^65^ ^]^ Perturbed Linear Mixing Model,^[^ ^66^ ^]^ Multivariate Curve Resolution (MCR),^[^ ^67^ ^]^ Augmented Linear Mixing Model,^[^ ^68^ ^]^ and Non‐negativity Matrix Factorization (NMF).^[^ ^69^ ^]^

When considering matrix‐based linear models, it is natural to consider PCA.^[^ ^58^ ^]^ However, PCA is not applicable for the HSI unmixing task. The reason lies in the orthogonality assumption of PCA's decomposed principal components (loadings and scores). In lyophilization HSI spectral unmixing, it is nearly impossible for spectral profiles from the HSI imaging system (such as NIR‐HSI and Raman‐HSI) to be orthogonal. Despite the limitation, PCA serves as a pre‐analysis tool for other spectral unmixing methods. For example, PCA helps estimate the appropriate number of components in MCR modeling and aids in exploration in lyophilization HSI data analysis. Similarly, Independent Component Analysis (ICA) does not perform well for lyophilization HSI spectral unmixing in many practical cases. ICA assumes statistically independent bilinear components, whereas pure spectral profiles in lyophilization HSI data rarely exhibit independence with each other, e.g., the chemical attributes in the lyophilization process may present high correlation. Nonetheless, ICA remains versatile for other modeling purposes in lyophilization HSI data analysis, such as HSI image classification and compression. Among the LMM‐based or LMM‐like models, MCR stands out as one of the most popular methods for HSI spectral unmixing. Similar to Equation 6, the MCR model can be written as

(7) $$X=DS^{⊤}+E$$

where **X** is the reconstructed raw HSI matrix, **D** is the decomposed concentration matrix corresponding to **A** in LMM equation, **S** ^⊤^ is the decomposed pure spectral profiles matrix corresponding to **B** in LMM equation, and matrix **E** contains the model residual. Unlike PCA, MCR has the capability to extract a pure spectral profile and concentrations while maintaining chemical meaning under a specific model constraint.^[^ ^70^ ^]^ For instance, instead of yielding orthogonal components, the non‐negativity constrained MCR model provides non‐negative pure spectral profiles and concentrations that aligns with real world scenarios in lyophilization HSI analysis. In contrast, PCA cannot guarantee that the decomposed components are chemically meaningful as they may include negative values. Beyond non‐negativity constraints, MCR offers flexibility in imposing additional model constraints–an essential advantage for HSI data analysis. For example, local rank constraints enhance the reliability of MCR solutions in HSI data analysis.^[^ ^71^ ^]^ The workhouse algorithm for fitting the MCR model is the alternating least squares algorithm (MCR‐ALS).^[^ ^72^ ^]^ MCR‐ALS starts with a determination of the number of components, followed by an initial guess on the loading and score matrices **D** and **S** ^⊤^. After a set of alternating least squares optimization steps on the estimates **D** and **S** ^⊤^, the model computation will end when the error change satisfies a predefined criterion. From that, the pure spectral profile and its abundance profile of different endmembers can be obtained. The simplicity, scalability, and chemically meaningful solutions provided by MCR‐ALS make it a useful tool for lyophilization HSI spectral unmixing. Regardless of whether MCR or other matrix‐based models such as NMF are used, they all operate by converting the raw lyophilization HSI third‐order data cube into a second‐order data matrix. Since HSI data inherently have a three‐way structure, straightforward unfolding may lead to loss of spatial information with respect to the relative positions of pixels.^[^ ^73^ ^]^ Furthermore, in many cases involving complex lyophilization tasks, unfolding HSI data into a matrix results in a significant increase in the number of variables along one of the matrix dimensions, posing computational challenges for matrix‐based models.

In recent years, tensor decomposition methods have been explored to address the limitation of matrix‐based HSI unmixing models. The CP (CANDECOMP/PARAFAC) model is one such tensor decomposition model used for lyophilization HSI spectral unmixing. In the CP model, lyophilization HSI data are considered as being in tensor format $\underline{X}$, which is then decomposed into a set of three‐way outer products in the case of a third‐order HSI tensor. The decomposition is expressed as

(8) $$\underline{X}=\underline{\hat{X}}+\underline{E}=\sum_{f=1}^{F}a_{f}\circ b_{f}\circ c_{f}+\underline{E}.$$

where $\underline{\hat{X}}$ denotes the tensor model, *F* is the tensor rank, and $\underline{E}$ corresponds to the residual. The third‐order vector (**a**_*f*_, **b**_*f*_, and **c**_*f*_) contains the *f*th column vector from the matrices **A**, **B**, and **C** respectively, which are derived from the third‐order HSI tensor. The CP model can be written in the matrix form as

(9) $$X_{I\times JK}=A{\left(C⊙B\right)}^{⊤}+E_{I\times JK}$$

where **X**_*I* × *JK*_ is the unfolding matrix of HSI tensor $\underline{X}$, and the dimensions of the decomposed matrices **A**, **B** and **C** are *I* by *F*, *J* by *F*, and *K* by *F*, respectively. The resulting CP format enhances model interpretability for lyophilization HSI data and facilitates handling the complex lyophilization analytic tasks. The decomposed matrices **A**, **B**, and **C** can characterize the spatial, spectral, and temporal/angle aspects in continuous lyophilization process. Recently, various tensor models have been investigated for performing HSI spectral unmixing tasks.^[^ ^73^ ^]^

**Figure 4:**
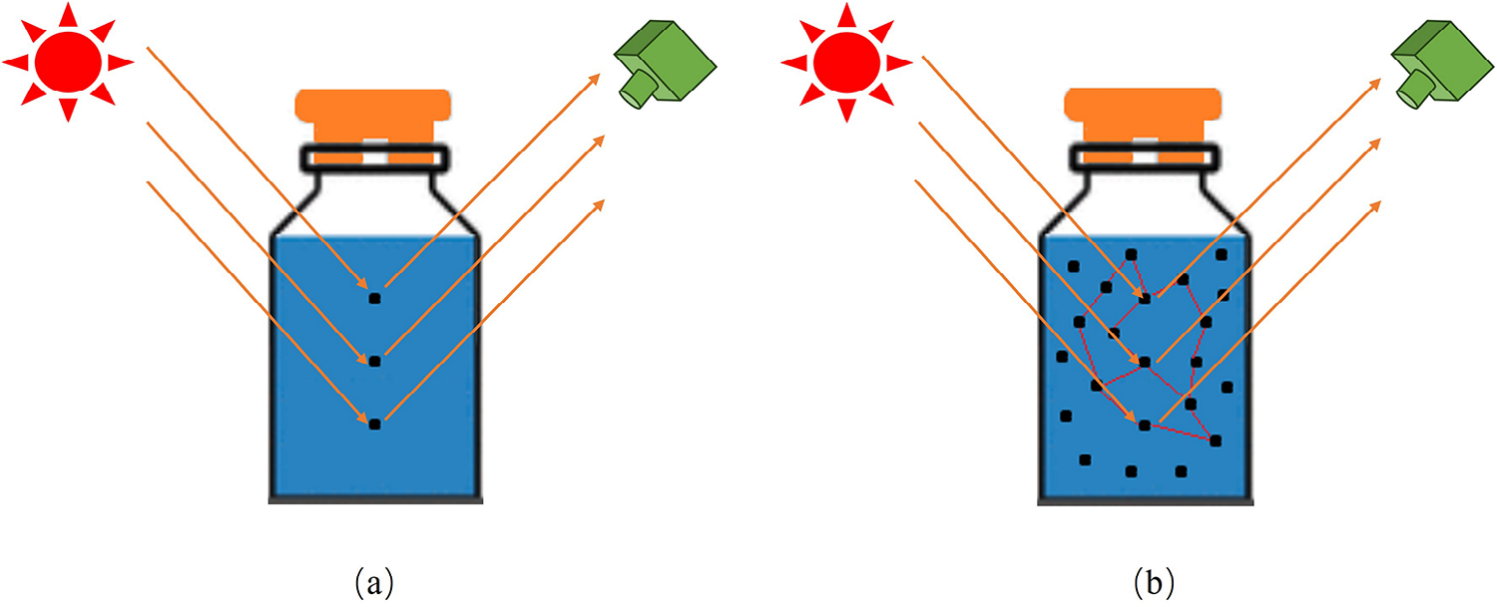
A graphical illustration on linear and nonlinear HSI mixture in lyophilization application caused by a) single and b) multiple scattering, respectively.

In practical lyophilization applications, physical interactions among different materials may exist in lyophilization HSI measurement scenes. For such cases, the assumption made by LMM‐like models that interactions between different endmembers are negligible falls short. Consequently, using linear models may lead to inaccurate and misleading HSI spectral unmixing results. Nonlinear unmixing challenges arise due to multiple scattering effects, which can be observed in scenarios involving multi‐layered materials lyophilization applications. A visual comparison between linear and nonlinear HSI measurement scenes in the lyophilization process is shown in **Figure** **4**. To address the nonlinear unmixing challenges, various nonlinear mixture models have been developed. These include polynomial mixing models,^[^ ^74^ ^]^ robust nonnegative matrix factorization,^[^ ^75^ ^]^ graphical models,^[^ ^76^ ^]^ neural network and kernel methods,^[^ ^77^ ^]^ and nonlinear low‐rank tensor factorization unmixing method.^[^ ^78^ ^]^ These nonlinear methods can be broadly classified into two categories. One category is physics‐based methods. These methods use mathematical tools to model the physical or optical interaction, such as polynomial mixing models. The other type of methods are data‐centered. These approaches derive results directly from the HSI data using machine learning techniques, such as neural network and kernel methods. Although nonlinear models currently receive much less attention than prevalent linear models, they are poised to gain increasing interest in the future due to the growing analytical demands posed by complex lyophilization products and processes.

### 3.3 HSI Regression and Classification

In biochemical engineering, such as in the pharmaceutical and food industries, constituent quantification is of major interest for process understanding and product quality analysis. Regression methods play an important role in constituents quantification of HSI measured objects in lyophilization applications. These regression methods can be broadly categorized into two types: linear models and nonlinear models. Multiple linear regression (MLR) is a typical linear model that calculates regression coefficients to model the linear relationship between a set of independent variables and a dependent variable.^[^ ^79^ ^]^ MLR has limitations when dealing with collinearity in the data. Collinearity is common in HSI analysis in lyophilization applications. For example, two chemicals may exhibit similar concentration profiles in HSI measurements. The collinearity can be addressed by partial least squares (PLS). Unlike MLR, PLS considers the covariance between the independent variable matrix and the dependent variable matrix by projecting them into a new space.^[^ ^80^ ^]^ PLS can be expressed as

(10) $$X=TP^{⊤}+E,\;\;Y=UQ^{⊤}+E,\;\;U=WT,$$

where **T** and **P** are scores and loadings of **X**, and **U** and **Q** are scores and loadings of **Y**. In the new space defined by **T** and **U**, the regression coefficients **W** can be obtained. The combination advantage of robust modeling on collinear data and multivariate linear regression makes PLS a consistently valuable tool for lyophilization HSI data regression analysis.

Although lyophilization HSI data are often linear in many cases due to the Beer–Lambert Law governing the spectral range,^[^ ^81^ ^]^ nonlinear relationships exist in some lyophilization HSI datasets. Nonlinear regression methods that have been developed for large HSI data analysis include nonlinear PLS,^[^ ^82^ ^]^ support vector regression (SVR),^[^ ^83^ ^]^ and artificial neural networks.^[^ ^84^ ^]^ A substantial dataset is typically required to fully leverage the advantages of specific nonlinear models, due to their increased degrees of freedom. The predictive accuracy of a regression model based on lyophilization HSI data depends on the quality of the calibration data, the number of representative samples, and preprocessing methods (e.g., variable selection). Experiments design aiming to provide sufficiently representative lyophilization HSI data and preprocessing procedures tailored to the spectral characteristics are useful for improving the performance of lyophilization HSI regression models. Removing data associated with frequency ranges containing spectral artifacts or lacking signal associated with the interests species also contributes to such improvements. Moreover, rigorous cross‐validation using independent datasets and repeated analysis using different calibration, validation, and testing splits is beneficial for avoid overfitting.^[^ ^85^ ^]^

In many cases, the regression models or their variants, are also employed for classification purposes. For instance, partial least squares‐discriminant analysis (PLS‐DA) is widely used to build classification models.^[^ ^86^ ^]^ Apart from PLS‐DA, other supervised classification methods can also be used in lyophilization HSI data analysis. Linear discriminant analysis and k‐nearest neighbor (k‐NN) are popular choices for supervised classification in HSI data analysis.^[^ ^87^ ^]^ Some recent methods proposed and tested in the context of HSI classification analysis include radial basis function neural network,^[^ ^88^ ^]^ deep support vector machine,^[^ ^89^ ^]^ convolutional neural networks,^[^ ^90^ ^]^ and Gaussian naive Bayes classification^[^ ^91^ ^]^ etc. The solutions of both linear and nonlinear HSI classification problems can be reached from the above‐listed methods. To address complex lyophilization HSI classification problems, ensemble strategies are recommended, which combine different classification methods to leverage the strengths of both linear and nonlinear classifiers. Spatial correlation among neighboring pixels in lyophilization HSI images has the potential of achieving precise classification, so called spectral‐spatial techniques. Mathematical morphology‐based methods are one type of spectral‐spatial techniques. For example, deep learning‐based extended morphology‐nonlocal capsule network method has been proposed for HSI classification in food analytic applications.^[^ ^92^ ^]^ Moreover, transfer learning based technique also demonstrates a significant performance in performing complex HSI image classification.^[^ ^93^ ^]^

Tensor methods show the potential for addressing the challenges posed by high‐order regression and classification tasks in lyophilization HSI data analysis. Unlike matrix‐based methods, tensor methods exploit the natural tensor structure of lyophilization HSI data during regression and classification modeling. Tensor methods have demonstrated advantages in achieving better model performance, even with a small number of training samples and significant spectral variations in the HSI data.^[^ ^94^ ^]^ One useful tensor model used for high‐order regression and classification purposes in HSI data analysis is the Tucker model,

(11) $$\underline{X}=\underline{S}\times_{1}A\times_{2}B\times_{3}C+\underline{E},$$

where $\underline{X}$ is the raw HSI tensor with dimension *I* by *J* by *K*; $\underline{S}$ is the tensor core array with dimension *F*_1_ by *F*_2_ by *F*_3_; and **A**, **B** and **C** are the decomposed matrix corresponds to each order, with the dimension *I* by *F*_1_, *J* by *F*_2_, *K* by *F*_3_, respectively. The Tucker model can be written in matrix form as

(12) $$X_{I\times JK}=AS_{F_{1}\times F_{2}F_{3}}\left(C⊗B\right)+E_{I\times JK}$$

where **X**_*I* × *JK*_ is the unfolding matrix of HSI tensor $\underline{X}$ and **S**_*I* × *JK*_ is also the matricization of tensor core array $\underline{S}$. The Tucker decomposition terms are widely used for classification purposes. For instance, a sparse tensor‐based classification method employs Tucker decomposition to extract joint spatial‐spectral tensor features while enforcing sparsity constraints on the tensor core array.^[^ ^95^ ^]^ Accurate and robust classification has been demonstrated for these extracted features. Another relevant tensor model is N‐way partial least squares (N‐way PLS), which extends PLS to third‐ and higher order tensors. The discriminative version, N‐way partial least squares‐discriminant analysis (NPLS‐DA), has been used for classifying tensorial HSI data. Using Tucker decomposition, the independent variables data in N‐way PLS^[^ ^96^ ^]^ is expressed as

(13) $$X_{I\times JK}=TS_{X}{\left(P_{3}⊗P_{2}\right)}^{⊤}+E_{I\times JK}$$

where **X**_*I* × *JK*_ is the unfolding matrix of HSI tensor $\underline{X}$, **T** is the score matrix of the first mode, **P_2_** and **P_3_** are the weight matrix of the second and third modes, **S**_*X*_ is the matricization of the tensor core array with the dimension of *F* by *F* by *F*, *F* is number of components, and **E**_*I* × *JK*_ is the matricization of residual tensor $\underline{E}$. Similarly, the dependent variables data in N‐way PLS is defined by

(14) $$Y_{I\times MN}=US_{Y}{\left(Q_{3}⊗Q_{2}\right)}^{⊤}+E_{I\times MN}$$

where the terms are defined in the same manner as in the independent variables equation. The regression coefficients **V** can be obtained from the expression **U** = **T** **V** + **E**. N‐way PLS and its variants have been applied and developed in the HSI literature for regression and classification tasks.^[^ ^97^, ^98^ ^]^ Additionally, the CP decomposition, a standard tensor model, has found applications in HSI data analysis for classification and regression purposes. Recent research introduces a generalized tensor regression model that leverages information from CP decomposition and extends multivariate labels ridge regression to construct a tensor classifier for HSI classification.^[^ ^99^ ^]^ Under the framework of mathematical morphology, the resulting terms from CP decomposition of HSI tensorial data are utilized for pixel‐wise classification in a low‐dimensional feature space. The CP‐based classification method is able to handle the multi‐modality structure inherent in HSI data.^[^ ^100^ ^]^ In summary, tensor‐based methods will offer new valuable insights for robust regression and accurate classification modeling of complex lyophilization HSI data.

### 3.4 HSI Data Fusion

Data fusion has emerged as a dynamic field within lyophilization HSI data analysis in recent years. Advances in instrumentation technology and ongoing developments in computational methods, such as chemometrics, now allow extraction of rich information from a single HSI analytical platform, yielding thousands of gigabytes of data in a short time. However, relying solely on data from a single HSI platform is insufficient for addressing all lyophilization challenges. For example, consider mass spectrometry imaging and Raman spectroscopy imaging. In Raman spectroscopy imaging, the signal intensity of a compound is directly related to its absolute concentration in the measured sample. Unfortunately, Raman spectroscopy techniques suffer from low molecular specificity and sensitivity, and are also susceptible to fluorescence interference.^[^ ^101^ ^]^ On the other hand, mass spectrometry imaging offers high molecular specificity and sensitivity due to its high mass resolution, making it suitable for detecting and analyzing a wide range of complex materials.^[^ ^102^ ^]^ However, the performance of mass spectrometry imaging can be influenced by the biochemical matrix. To address these limitations, fusing Raman spectroscopy imaging and mass spectrometry imaging data provides a complementary combination of essential biochemical information. HSI data fusion enhances the ability to gain profound insights from complex lyophilization materials and processes, thereby improving the application of HSI techniques for analytic, characterization, monitoring, and control in lyophilization contexts. Data fusion in lyophilization HSI involves integrating information from various sources to enhance the overall problem‐solving capability. For example, the fusion of image data from different HSI techniques platforms such as Vis‐NIR‐HSI and NIR‐HSI multi‐modal hyperspectral images fusion,^[^ ^103^ ^]^ and the fusion of image data of HSI and other analytic technologies such as HSI and MSI.^[^ ^104^ ^]^ In lyophilization applications, these types of HSI data fusion have been useful in leveraging the combined advantages of different analytic techniques to better characterize and understand complex lyophilization materials and processes.

**Figure 5:**
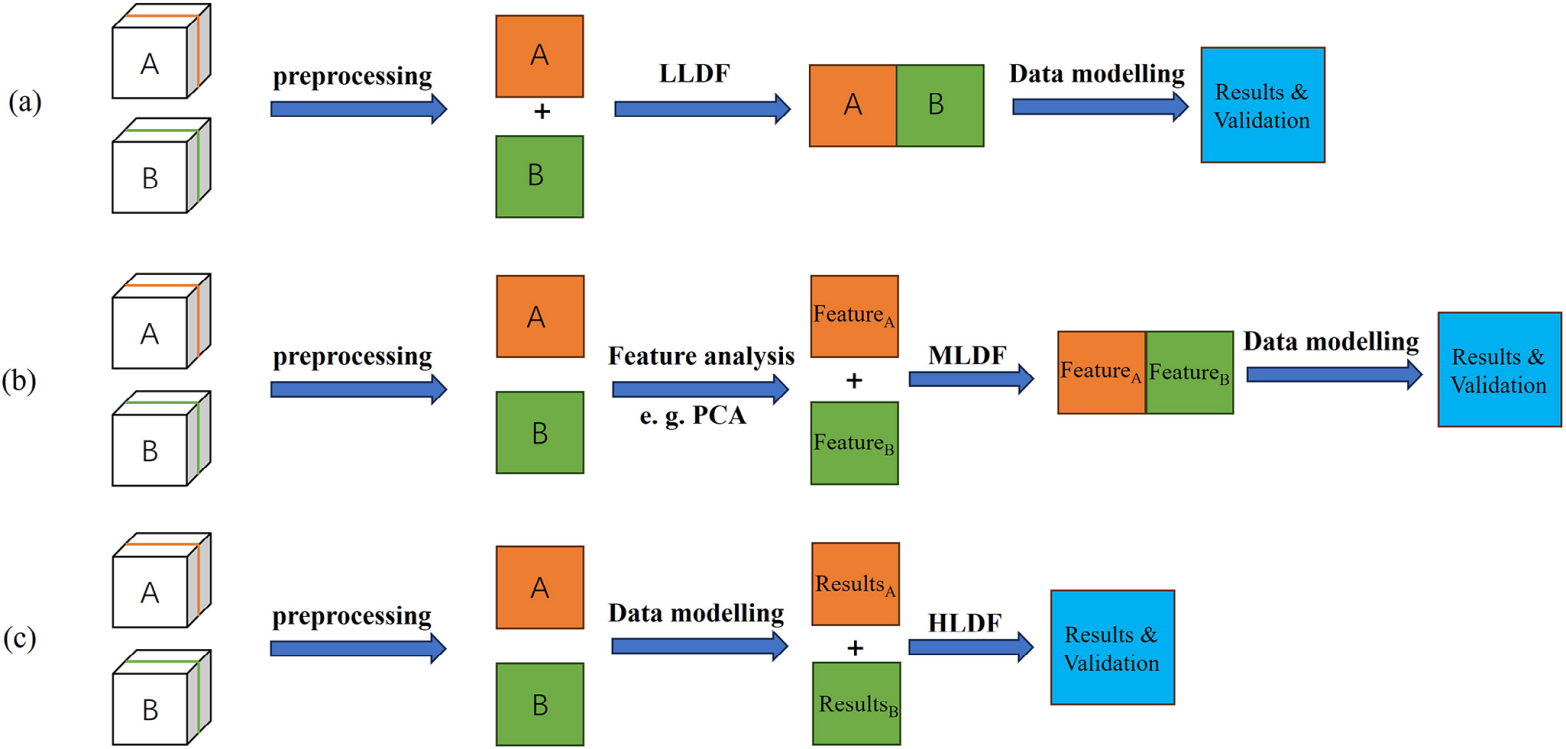
An illustration of different levels of lyophilization HSI data fusion and the data modeling workflow: a) LLDF, b) MLDF, c) HLDF.

Data fusion in lyophilization HSI involves three distinct levels:^[^ ^105^ ^]^ low‐level data fusion (LLDF), middle‐level data fusion (MLDF), and high‐level data fusion (HLDF). In LLDF, HSI data from different sources are directly merged, and the combined data undergo joint analysis using chemometrics and other mathematical or statistical methods. LLDF is an intuitive approach for lyophilization HSI data analysis. At the MLDF level, features extracted from models based on different source HSI data are employed for joint analysis. For example, principal component features obtained through PCA on lyophilization HSI data can be used for MLDF analysis. HLDF occurs at the decision layer and is determined by the specific modeling purposes. For example, in prediction analysis, individual prediction models are built for each lyophilization HSI dataset. The results from these models are then fused for the final prediction analysis. However, HLDF is rarely used in chemistry or chemical engineering.^[^ ^106^ ^]^ The level of HSI data fusion directly determine the used validation methods. In the case of cross‐validation, the validation is done in a single LLDF model which is the sole model in LLDF HSI analysis, while validations are performed in each model in MLDF and this leads to a set of cross‐validation analysis since each HSI dataset has its own model in MLDF and each model have their own model validation. Different levels of lyophilization HSI data fusion and its data modeling workflows are presented in **Figure** **5**.

Various methods have been explored and employed for HSI data fusion modeling. Among these, matrix‐based approaches have dominated HSI data fusion analysis for an extended period. Methods such as MCR and NMF are commonly used for analyzing fused HSI data, although they operate differently in HSI data fusion modeling. For instance, the MCR model typically operates on the unfolded pixel spectral matrix of an individual HSI data cube, aiming to unmix the spectral components. However, when dealing with fused HSI data, the MCR model must be applied to a combined multiset of HSI datasets. In other words, different HSI data cubes are interconnected, and must share a common dimension—such as spectral, spatial, or pixel dimensions—in the fused multiset HSI data.^[^ ^107^ ^]^ When fusing images from the same HSI platform, they naturally share the common spectral dimension, as the spectral dimension is the same for HSI images coming from the same HSI platform. In cases where images come from the same sample but different HSI platforms, they typically share the common pixel dimension. For data fusion within the same HSI platform, the bilinear matrix‐based model can be expressed as

(15) $$\left[X_{1}\;X_{2}\;\cdots \;X_{n}\right]=\left[D_{1}\;D_{2}\;\cdots \;D_{n}\right]S^{⊤}+\left[E_{1}\;E_{2}\;\cdots \;E_{n}\right]$$

where [**X_1_** **X_2_** … **X_n_**] is the pixel spectral matrix containing the submatrices from each image set, [**D_1_** **D_2_** … **D_n_**] is the concentration profile of the constituent in each image set, **S** ^⊤^ denotes the common pure spectral signature and [**E_1_** **E_2_** … **E_n_**] is the big residual matrix composed by submatrices representing the model residuals associated with each image set. This type of HSI fusion modeling is applicable for lyophilization analysis. When employing an HSI device to analyze and monitor the lyophilization process in pharmaceutical/food manufacturing, data from different stages of the lyophilization process are fused together. Building a data fusion model allows us to understand the evolution of important attributes and achieve online quality control during the lyophilization process. When constructing models using fused data from different HSI platforms, the bilinear matrix‐based model needs to be adjusted to

(16) $$\left[X_{1}\;X_{2}\;\cdots \;X_{n}\right]=D\left[S_{1}^{⊤}\;S_{2}^{⊤}\;\cdots \;S_{n}^{⊤}\right]+\left[E_{1}\;E_{2}\;\cdots \;E_{n}\right]$$

where [**X_1_** **X_2_** … **X_n_**] is the multiset data consisting of the images from the same sample but different HSI platforms, **D** is the concentrations profile of the constituent in the samples, $\left[S_{1}^{⊤}\;S_{2}^{⊤}\cdots \;S_{n}^{⊤}\right]$ is the large spectral signature matrix containing the pure spectral of constituents in the image of each HSI analytic technique, and [**E**_**1**_ **E**_**2**_ … **E**_**n**_] is the big residual matrix composed by submatrices representing the model residuals associated with each HSI analytic technique. When analyzing fused lyophilization data from different types of HSI, the spectral wavelengths and signal characteristics, such as intensity, vary across different analytical techniques. Therefore, employing appropriate preprocessing methods–such as scaling and centering techniques–on various types of lyophilization HSI data plays an important role in implementing data fusion modeling and analysis. Additionally, it is essential to address potential differences in spatial resolution when using bilinear matrix‐based models for fused lyophilization HSI data analysis. Image matching algorithms are useful for handling spatial direction difference challenges in HSI data fusion modeling.^[^ ^108^ ^]^ In cases where significant spatial resolution differences exist among different HSI techniques, advanced methods such as multi‐block modeling or extended matrix‐based models with multiple objective functions are recommended. For an in‐depth discussion of these methods, the specific references are suggested.^[^ ^109^, ^110^ ^]^

**Figure 6:**
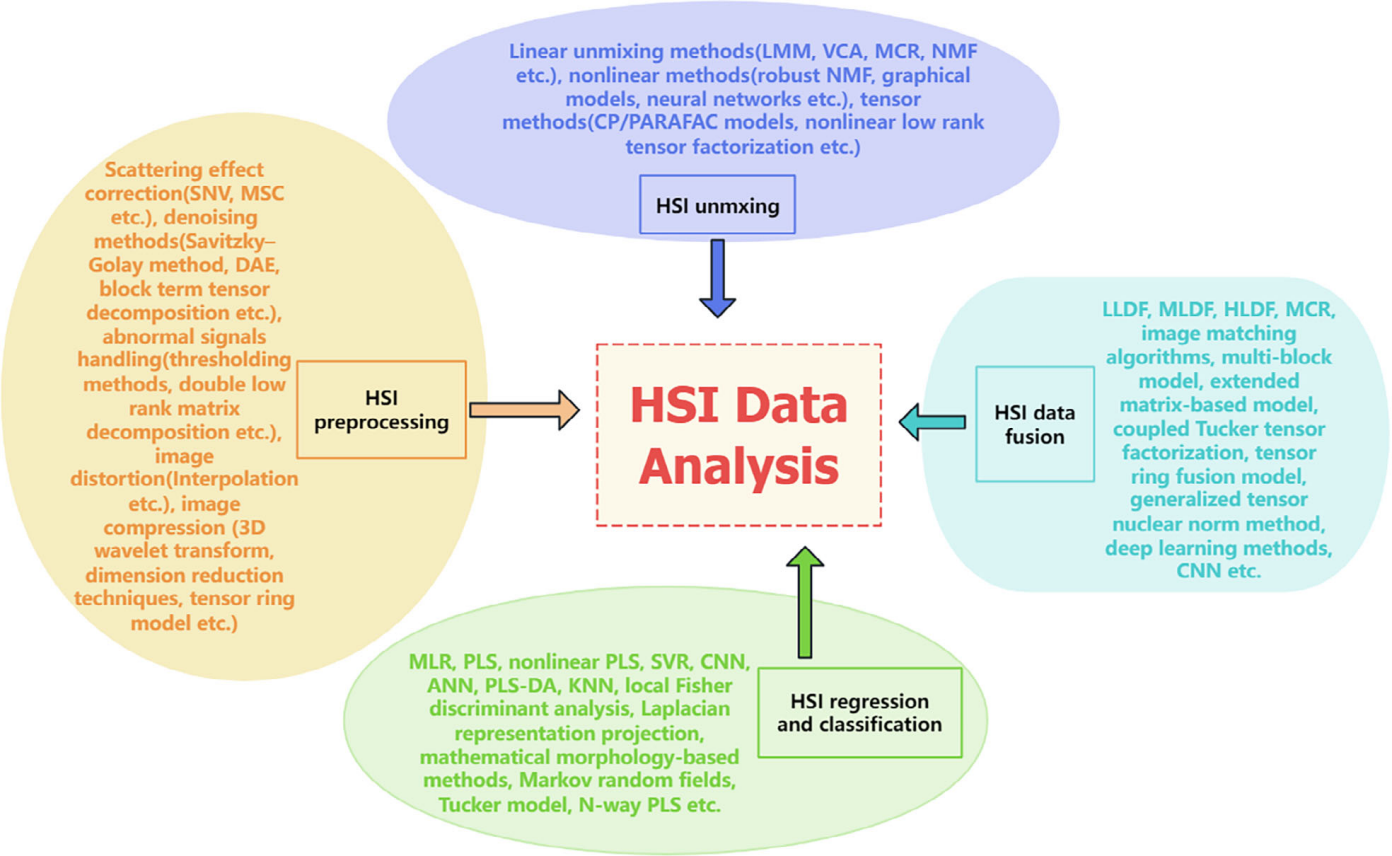
A graphical illustration on the typical data‐driven modeling strategies for lyophilization HSI data analysis.

Tensor‐based and deep learning‐based approaches have recently been developed for HSI data fusion analysis. These methods not only address challenges related to high dimensionality and large data volumes in HSI data fusion but also have the potential of enhancing model interpretability and expert analysis in practical lyophilization applications. A coupled Tucker tensor factorization method has been proposed for handling inter‐image variability in HSI‐MSI data fusion analysis.^[^ ^111^ ^]^ This Tucker tensor‐based approach handles spatial and spectral variations with lower computational cost than prior state‐of‐the‐art methods. A novel tensor ring fusion model using Bayesian sparse learning techniques has been developed for HSI‐MSI image fusion analysis.^[^ ^112^ ^]^ The proposed Bayesian probabilistic tensor framework automatically determines the true latent rank in the fusion model, eliminating the need for manual selection. Deep learning‐based methods can efficiently learn fusion features from large image datasets. Nonlinear functions and multiple layers are leveraged to achieve high‐precision HSI fusion.^[^ ^113^ ^]^ An unsupervised deep learning network has been proposed for HSI‐MSI fusion modeling that combines a tensor decomposition model with a deep learning network, to learn a shared code tensor and use it to infer high‐resolution HSI images.^[^ ^114^ ^]^ An enhanced blind HSI‐MSI image fusion method using a deep learning framework has been developed that is capable of achieving better spatial and spectral accuracy by applying modified spectral normalization to the network weights.^[^ ^115^ ^]^ In addition to HSI‐MSI image fusion analysis, tensor‐ and deep learning‐based methods have been used for fusion modeling in other types of HSI techniques. Tensor‐based data fusion methods show potential for prediction and regression analysis across different types of HSI data fusion.^[^ ^116^ ^]^ Convolutional neural networks have also been explored for analyzing fused data from Vis‐HSI and NIR‐HSI, with learned features used for classification analysis.^[^ ^117^ ^]^ A drawback of deep learning methods for HSI image fusion analysis is their low generalization ability. Trained models tend to be highly dependent on the specific type of input HSI data, limiting their wider applicability. In contrast, tensor‐based methods do not suffer from this limitation in most cases. Continued advances in HSI data fusion models and methods will undoubtedly further promote the application of HSI for solving complex problems in lyophilization‐related contexts. **Figure** **6** provides a graphical illustration of typical data‐driven modeling strategies for lyophilization HSI data analysis, including HSI preprocessing, unmixing, regression, classification, and data fusion, being discussed in this article.

## 4 Applications in Lyophilization

Recall that lyophilization consists of three main steps, namely (1) freezing, (2) primary drying, and (3) secondary drying. During freezing, it is common to monitor the product temperature (and sometimes crystal structure) to ensure that the liquid is completely frozen before proceeding to the drying step. In primary drying, monitoring the temperature is critical because the maximum product temperature should not exceed the glass transition and collapse temperatures. Besides, the sublimation flux is sometimes measured to ensure that the sublimation process is complete. In secondary drying, the most important parameter is the residual moisture (bound water), which directly affects the final product quality (stability). Existing methodologies for analyzing lyophilized samples predominantly rely on offline techniques,^[^ ^2^ ^]^ which are often destructive in nature, involving (typically labor‐intensive^[^ ^2^ ^]^) physical and chemical analyses.^[^ ^1^ ^]^ Measuring the temperature is relatively simple, e.g., with the use of intrusive thermocouples. The product moisture is analyzed using the Karl‐Fischer (KF) titration^[^ ^2^, ^4^ ^]^ or thermogravimetry^[^ ^8^ ^]^ approaches. The sublimation flux is usually measured by the pressure rise test.^[^ ^4^ ^]^ These methods are characterized by a substantial time delay between measurement and process. To address the limitations of traditional chemical and physical methods, researchers have increasingly focused on using chemometrics and HSI techniques.

### 4.1 Pharmaceutical Manufacturing

In pharmaceutical manufacturing, there are several critical quality attributes (CQAs) that have to be monitored and controlled to ensure efficacy and stability and satisfy regulatory requirements. The ability to extract spatial information in real time, which cannot be achieved by various traditional process analytical technology tools, allows the gaining of more insights about the products and processes, motivating recent interests in employing HSI in pharmaceutical applications. This spatial information is even more beneficial for processes such as lyophilization that have strong associated heterogeneity. For lyophilization, HSI can be used to provide additional information about some quantities that are commonly monitored by many existing sensors, e.g., moisture content,^[^ ^36^ ^]^ or study more complicated phenomena and properties that conventional techniques might not adequately capture, e.g., solid‐state transformation,^[^ ^118^ ^]^ which is discussed below.

#### 4.1.1 Determining Residual Moisture and Temperature

**Figure 7:**
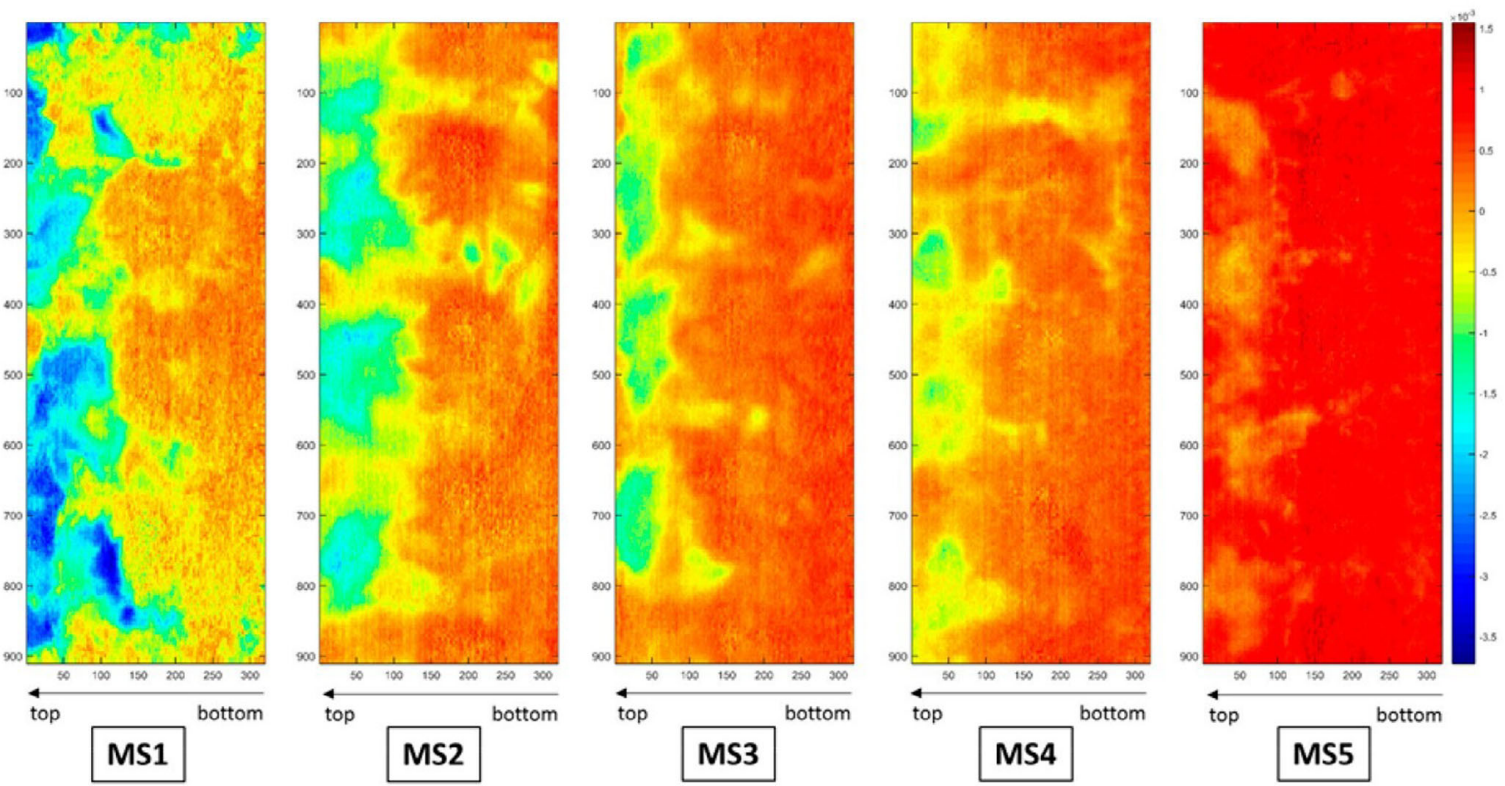
Reconstructed images of five different mannitol‐sucrose samples MS1 to MS5 representing variations from low (blue) to high (red) water levels. Reproduced with permission from Brouckaert et al.^[^ ^2^ ^]^ Copyright 2018, American Chemical Society.

Residual moisture (aka water content, bound water concentration) and temperature directly affect the quality of the final product and can be measured using many standard techniques such as Karl‐Fischer titration or NIR spectroscopy, but those approaches do not provide any information on spatial heterogeneity. With HSI, spatiotemporal evolution of the residual moisture and temperature can be studied. For example, NIR‐HSI has been used to investigate the distribution of water during conventional lyophilization of unit doses.^[^ ^36^ ^]^ The image data were processed via PCA, with the PLS regression model developed from Karl‐Fischer titration data. NIR‐HSI was shown to quickly and accurately determine the water content within 96 vials containing mannitol, sucrose, lysozyme, and bovine serum albumin (BSA). A similar study^[^ ^2^ ^]^ used NIR‐chemical (NIR‐CI) imaging for determination of the water content of lyophilized products during continuous lyophilization via spin freezing. For mannitol‐sucrose samples, NIR‐CI combined with PCA and PLS was used to reveal the spatial heterogeneity in moisture content between different vials and also within each vial (see **Figure** **7**), with the results validated using Karl‐Fisher titration. IR‐HSI has been used for online monitoring of the spatiotemporal evolution in human serum vials during lyophilization.^[^ ^119^ ^]^ The technique was shown to be a versatile and powerful tool for online monitoring of lyophilization.

#### 4.1.2 Investigating Solid‐State Properties

In lyophilization of pharmaceutical products, the formulation consists of many components; for example, lyophilization of mRNA vaccines entails mRNA, lipid nanoparticles, lyoprotectants (e.g., Tris), and cryoprotectants (e.g., sucrose).^[^ ^12^ ^]^ HSI can be used to analyze various properties of the complicated solid structures which impact the final product quality.

NIR imaging has been employed to study the crystallization of sucrose in multiple lyophilized sugar‐protein samples,^[^ ^36^ ^]^ a process that affects the stability of proteins. By having the reference spectra of amorphous and crystalline sucrose, data from NIR images were used to identify the time when crystallization starts, the minimum sucrose concentration where crystallization appears, and the effects of protein on inhibiting sucrose crystallization. Since the stability of the final product and reconstitution time could be influenced by crystalline components, SNV and augmented MCR modeling were built on NIR hyperspectral images to explore the spatial distributions of β‐ and δ‐mannitol crystals in the vials during lyophilization.^[^ ^2^ ^]^ The spatial distributions varied between different vials and various positions in the same vial, which was attributed to spatial variations in the freezing rate and temperature.^[^ ^2^ ^]^ Temperature‐controlled Raman imaging has been used to examine the effects of annealing on the polymorphic forms of mannitol at various locations within lyophilized samples.^[^ ^120^ ^]^ Raman maps, constructed using PCA scores and Linear Combination of Elements (LCE) analysis, illustrate the distribution and transformation of polymorphic forms across the sample.

Raman mapping has been implemented to study the formation of complexes with cyclodextrins that entails furazolidone (FZD) and β‐cyclodextrin (β‐CD) or hydroxypropyl‐β‐cyclodextrin (HP‐β‐CD) prepared by lyophilization and kneading.^[^ ^121^ ^]^ Data from Raman images were analyzed and used to indicate that the most effective interactions can be obtained from the compounds prepared by lyophilization with a molar ratio of 1:2 (drug:CD), agreeing with past observations in the literature. A more recent study by Ref. [118] employed Raman mapping to detect solid‐state transformations, i.e., changes in matrix crystallinity, in the lyophilized iburofen solution. Raman spectra were obtained as the average of two experimental spectra at each sample point, with the most representative spectra selected for further analysis. The selected spectra were truncated to reduce noise, and PCA was subsequently used to identify significant spectral features. Correlation analyses between Raman spectra and off‐line X‐ray powder diffraction (XRPD) measurements were then conducted using PLS regressions. Results showed that the proposed technique can differentiate between samples with different amount of iburofen and mannitol, and also identified the time when transformations of iburofen and mannitol take place.

#### 4.1.3 Studying Bioprotection

Stabilizers or lyoprotectants—such as polyols, amino acids, and polymers—are added to pharmaceutical formulations, along with other excipients and buffering salts, to improve protein stability during lyophilization. Ice formation during freezing concentrates these excipients in the remaining liquid. Phase separation may occur if the excipients and proteins at the freeze concentration show thermodynamic incompatibilities and unfavorable molecular interactions.^[^ ^122^, ^123^, ^124^, ^125^ ^]^ When proteins are separated from their stabilizers, they can experience significant physical and chemical degradation. Detecting phase separation in the absence of crystallization and the presence of multiple amorphous phases is challenging using conventional techniques, such as scanning electron microscopy (SEM), differential scanning calorimetry (DSC), polarized light microscopy (PLM), and X‐ray powder diffraction (XRPD).^[^ ^5^, ^126^ ^]^ Alternatively, HSI has been shown to be an effective alternative for evaluating the homogeneity and phase separation of biopharmaceutical formulations containing stabilizing excipients.^[^ ^5^, ^126^, ^127^, ^128^ ^]^

To address the challenges in using SEM and DSC in detecting phase separation, NIR imaging has been used to evaluate the homogeneity of freeze‐dried protein‐stabilizer (lysozyme‐trehalose) mixtures.^[^ ^127^ ^]^ The raw reflectance spectra were converted into absorption spectra and normalized, and then Savitsky‐Golay filters were applied to calculate the second derivative of each spectrum. Analysis with these preprocessed spectra indicated that correlation coefficient mapping and PLS regression provide clearer contrasts for investigating spatial heterogeneity. Through tests of the mixtures with different ratios, NIR imaging combined with the correlation coefficient or PLS provided a potentially more detailed analysis of phase separation in lyophilized formulations. Raman mapping has been used to identify amorphous phase separation in freeze‐dried protein formulations.^[^ ^126^, ^128^ ^]^ Various mixtures – including Polyvinylpyrrolidone and Dextran, Ficoll and BSA, and trehalose and lysozyme – at different weight ratios were lyophilized and analyzed using Raman mapping, DSC, and NIR. The SNV method was used for processing the Raman data and the root mean square of the weighted deviations in the peak intensity at each point across the map was the indicator of the compositional deviations. The results from Raman mapping closely correlated with those from DSC analysis. Another study of Raman mapping analysis analyzed the impact of instrument parameters such as collection aperture, accumulation time, and line map length on the detection accuracy of phase separation in freeze‐dried protein formulations.^[^ ^5^ ^]^ The proposed Raman line‐mapping protocol reduced data collection time by 80% while ensuring validated detection capabilities.

**Figure 8:**
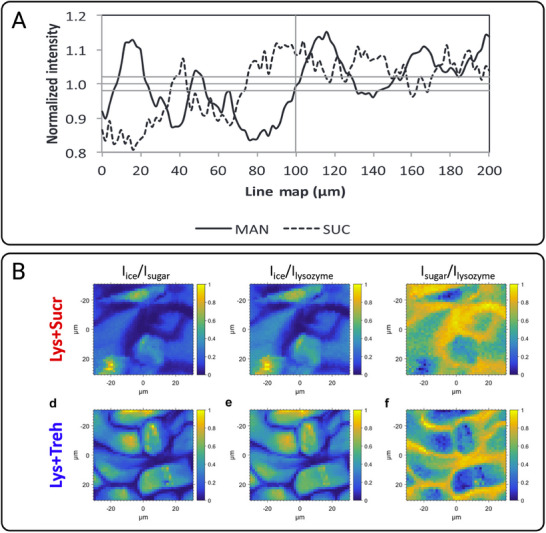
A) A line map showing the normalized Raman intensities (at 875 and 833 cm^−1^) of D‐mannitol (MAN) and sucrose (SUC) in a 1:2 wt% formulation.^[^ ^3^ ^]^ Reproduced with permission from Forney‐Stevens et al.^[^ ^3^ ^]^ Copyright 2014, Elsevier. B) Raman maps of the effect of the ratio of lysozyme to sugar (sucrose and trehalose) on freeze‐dried formulations after the freezing stage.^[^ ^129^ ^]^ Relative integrated intensity ratios are shown: Regions rich in ice are poor in disaccharide. Trehalose separates better than sucrose from the ice phase. Reproduced with permission from Starciuc et al.^[^ ^129^ ^]^ Copyright 2020, Elsevier.

Raman mapping has been used to investigate the mechanisms underlying protein stabilization by trehalose during freeze‐drying and the enhanced bioprotective properties of trehalose facilitated by glycerol.^[^ ^130^ ^]^ Throughout each stage of the freeze‐drying process, the interactions between water, trehalose, and lysozyme were analyzed. Polynomial function analysis and normalization methods were applied to all spectra collected during the mapping. Raman signatures of ice crystals, trehalose, and lysozyme in the samples suggested that the inclusion of glycerol contributes to a more uniform distribution of chemical species after freezing. Trehalose's primary bioprotective effect was observed during the primary drying stage; adding glycerol reduced the effect while significantly enhancing bioprotection during the secondary drying stage by forming stronger glycerol‐trehalose H‐bonds. The spectral range of 80–1900 cm^−1^ in Raman images has been analyzed to evaluate the bioprotective efficiency of sucrose and trehalose in lyophilization.^[^ ^129^ ^]^ Raman images acquired after the freezing stage showed that sucrose exhibited a more spatially homogeneous distribution with lysozyme than trehalose, suggesting that sucrose provides a larger bioprotective effect than provided by lysozyme during the primary drying stage (see Figure 8). That is, pronounced phase separation within the lysozyme formulation in the presence of trehalose makes the lysozyme more vulnerable to interaction with the ice surface. The Amide I band in Raman spectra was analyzed to investigate protein denaturation. The peak of the Amide I band was determined by fitting Raman spectra with Gaussian functions, the 1500–1800 cm^−1^ spectral regions of which were baseline‐corrected. Shifts of the Amide I band toward higher frequencies after ice sublimation from all 1861 samples indicated alterations in the secondary structure of lysozyme. Additional shifts of the Amide I band were observed after secondary drying, indicating a further structural alteration of the lysozyme.

#### 4.1.4 Exploring Enzyme Immobilization

HSI has been used to investigate enzyme immobilization for biocatalysis, which has been increasingly used in pharmaceutical manufacturing.^[^ ^22^, ^131^ ^]^ Raman‐HSI was applied to lyophilized pantothenate kinase (PanK), which was immobilized to acrylamide‐based and methacrylate‐based resins.^[^ ^22^ ^]^ By using multivariate analysis in the form of PCA, Raman‐HSI with two different excitations, namely 532 and 785 nm, was able to resolve four distinct chemical species: the enzyme PanK, resin (acrylamide), buffering agent (Bis‐Tris), and glass substrate. Optimal instrumental parameters were identified, where 785‐nm excitation was found to perform better. A similar study^[^ ^131^ ^]^ investigated the immobilization of PanK using Raman mapping but instead relied on unsupervised machine learning in the form of NMF. By using Raman mapping with NMF, the technique resolved, both spatially and spectrally, the chemical species distributions, including PanK, both acrylamide and methacrylate resins, the glass substrate, adhesive glue, and Bis‐Tris, agreeing with the corresponding optical imaging results. These insights are useful for biocatalytic process development in pharmaceutical manufacturing.^[^ ^22^, ^131^ ^]^

#### 4.1.5 Other Applications and Possibility

**Figure 9:**
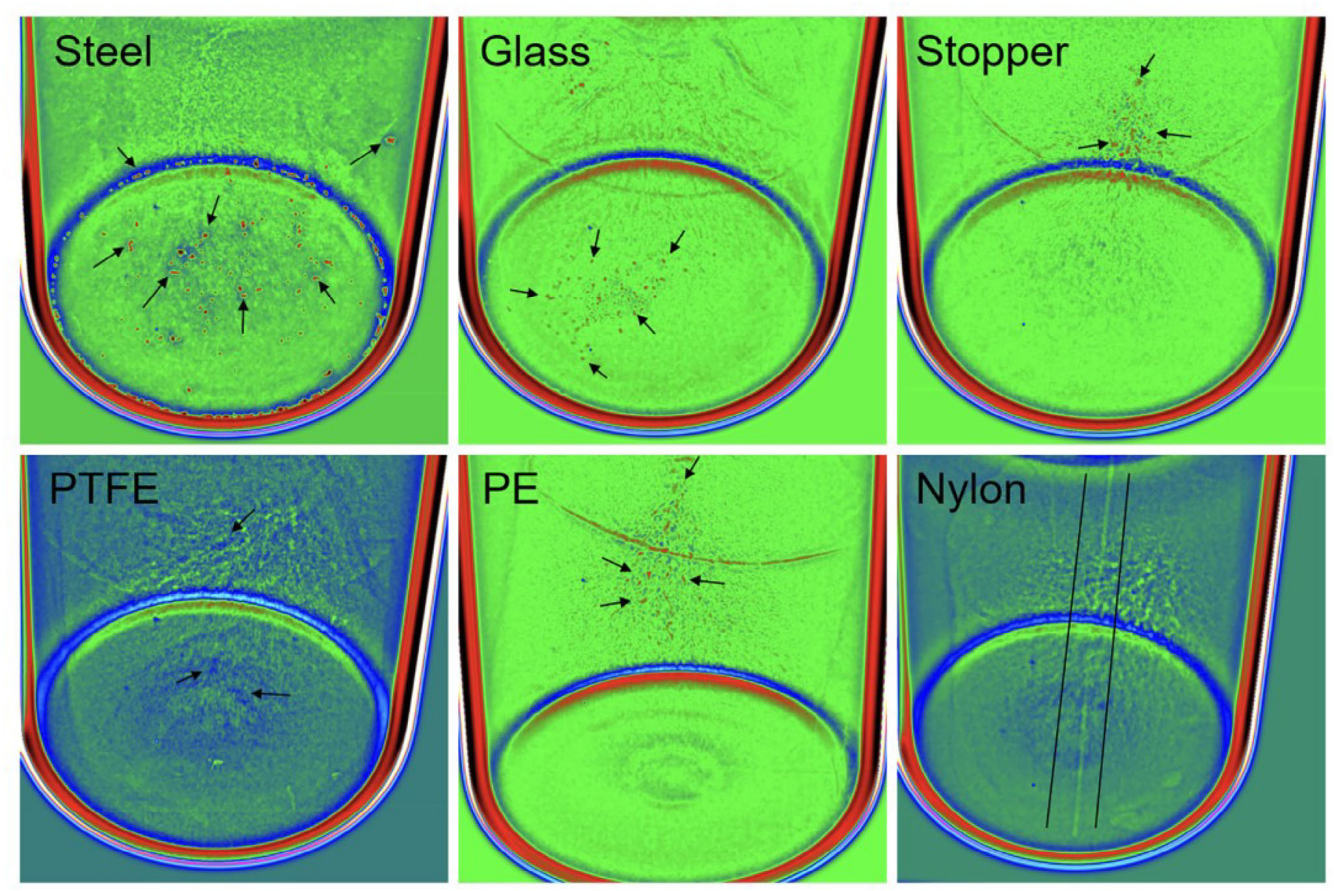
X‐ray images of vials with various particulates, namely steel, glass, stopper, polytetrafluoroethylene (PTFE), polyethylene (PE), and nylon. All spiked particulates in the vials were successfully detected by the X‐ray imaging technique. Reproduced with permission from Sacher et al.^[^ ^31^ ^]^ Copyright 2021, Elsevier.

HSI has potential value in a wide variety of applications. This section summarizes some other available and potential applications of HSI in pharmaceutical manufacturing that are not discussed above. One promising application is to detect foreign or undesirable substances/particulates in the lyophilization system.^[^ ^132^, ^133^ ^]^ Some imaging techniques, e.g., X‐ray imaging,^[^ ^31^ ^]^ were demonstrated to detect various particulates, including glass, steel, polymers, and organic particulates, in the lyophilized product prepared from human serum albumin. The technique could successfully identify steel and glass down to sizes of 80–100 µm, stoppers and polymers promising larger than about 160 µm, and organic particulates that expand in one dimension, e.g., hair and nylon strings (see **Figure** **9**). The only limitation is associated with the low contrast of some organic substances and small glass particles. The ability to detect particulates could be highly beneficial for the future of lyophilization, which is moving to continuous manufacturing. In continuous lyophilization, vials and products are usually moved continuously or spun (e.g.,^[^ ^134^ ^]^) which could increase the possibility of having small particulates in the system. Another interesting application is to study the pattern of a sublimation front/interface, e.g., using X‐ray imaging.^[^ ^135^ ^]^ Measuring the sublimation front is not easy with standard sensors/techniques implemented in many existing lyophilization systems, and hence it is typical to rely on measurement of product temperature or sublimation flux (e.g., via the pressure rise test) for monitoring of primary drying.^[^ ^4^, ^136^ ^]^ Being able to identify the sublimation front is valuable because it best represents the evolution of sublimation and hence could be a more accurate way to determine the end point of primary drying. Given that HSI gives complete spatial data of the product, extracting information about the sublimation front should be possible. A summary of the typical lyophilization applications of HSI in pharmaceutical field is presented in Table 1.

### 4.2 Food and Other Biologically Related Applications

Lyophilization has shown to be an effective technique for removal of water in the food industry with high‐quality final products. By eliminating liquid water and operating at low temperatures, this food drying process minimizes the risk of physiochemical changes in the food while preventing enzymatic and microbial deterioration.^[^ ^137^ ^]^ Although microwave vacuum‐drying is faster and hot air drying is more cost‐effective, the superior preservation of the quality and nutritional content of products makes freeze‐drying widely used in the food industry (Ref. [138]). The increased public demand for high‐quality food has driven the advancement in quality inspection of lyophilized food products.^[^ ^139^ ^]^ Whereas traditional methods such as chromatography and biotechnological tools, and recent non‐destructive techniques such as acoustic methods, have been developed and applied (^[^ ^139^ ^]^), these approaches often require complex sample processing and skilled technical staff or are unable to fully quantify the spatial heterogeneity inherent in food products. In contrast, HSI is a robust alternative by providing rapid spatial and spectral information from samples through a combination of imaging and spectroscopic techniques. The spectral data acquired at every HSI‐scanned point contains the physical and chemical properties of the sample, facilitating the assessment of the impact of a set of factors such as genetic diversity, climatic conditions, and agricultural practices.^[^ ^140^ ^]^ Thus, HSI has been used in quality inspection and control of various lyophilized food products including fish,^[^ ^6^, ^141^ ^]^ vegetables,^[^ ^32^, ^142^ ^]^ fruit,^[^ ^140^ ^]^ mushrooms,^[^ ^137^, ^143^ ^]^ cheese,^[^ ^144^ ^]^ and tea.^[^ ^145^ ^]^

#### 4.2.1 Determining Residual Moisture

Moisture content is a critical factor in determining the stability and quality of lyophilized food products. High residual moisture content in these products can facilitate the growth and reproduction of microorganisms, resulting in quality decay and deteriorative reactions.^[^ ^146^, ^147^ ^]^ To minimize these adverse effects and preserve the quality, flavor, and nutritional value of the food, it is essential to closely monitor the moisture level in these freeze‐dried products.

To assess the moisture content in grass carp (*Ctenopharyngodon idella*) slices subjected to various freeze‐drying durations, hyperspectral images were generated from Vis‐NIR spectroscopy.^[^ ^6^ ^]^ PLS with leave‐one‐out cross‐validation was used to reduce the high‐dimensional data to nine influential wavelengths, which are associated with the largest absolute regression coefficients for moisture content prediction. The results indicated that PLSs based on pretreated data compromises the prediction accuracy. A map of the spatiotemporal evolution of moisture content throughout the drying process across the grass carp slices was generated from the spectra and the regression coefficients. HSI has also been used to evaluate the moisture content in freeze‐dried shiitake mushrooms,^[^ ^137^ ^]^ where spectra of 19 wavelength regions from the UV (405 nm) to NIR (970 nm) were acquired. Three multivariate models, PLS, backpropagation neural network (BPNN), and least squares‐support vector machines (LS‐SVM), were compared for residual moisture estimation in the freeze‐dried mushrooms. Their findings indicate that LS‐SVM provides the highest accuracy in predicting water fractions in the samples. Another study collected a 3D hyperspectral cube with spatial dimensions of 816×540 pixels, and a spectral dimension of 616 nm wavelengths, to assess moisture content for the *Agaricus bisporus* mushroom.^[^ ^143^ ^]^ The SVM model, with MSC for data pretreatment and stability competitive adaptive reweighted sampling (SCARS) for key wavelength extraction, achieved good accuracy in moisture content prediction during the freeze‐drying process of *Agaricus bisporus*. Their moisture distribution map shows that the moisture content at the edge is lower than that at the core, indicating preferential sublimation at the sample edge. A study for a different food product analyzed the NIR region, which encompasses various fundamental molecular vibrations such as C‐H, N‐H, and O‐H, in chili peppers.^[^ ^148^ ^]^ The successive projections algorithm (SPA) had higher performance than competitive adaptive reweighted sampling (CARS) and genetic algorithm‐partial least squares (GA‐PLS) methods in managing redundancy and collinearity in the spectral data. Based on the most influential wavelengths identified by SPA, extreme learning machine (ELM) models achieved more accurate predictions of moisture content than PLS and LS‐SVM.

#### 4.2.2 Inspecting Quality and Safety Attributes

HSI has been used to assess the functional components and physical and chemical properties that serve as valuable quality indicators in freeze‐dried food products. These examples illustrate the potential for predicting key functional components in freeze‐dried food products, demonstrating significant industrial potential. This non‐invasive method provides a faster alternative to more time‐consuming and invasive techniques

**Figure 10:**
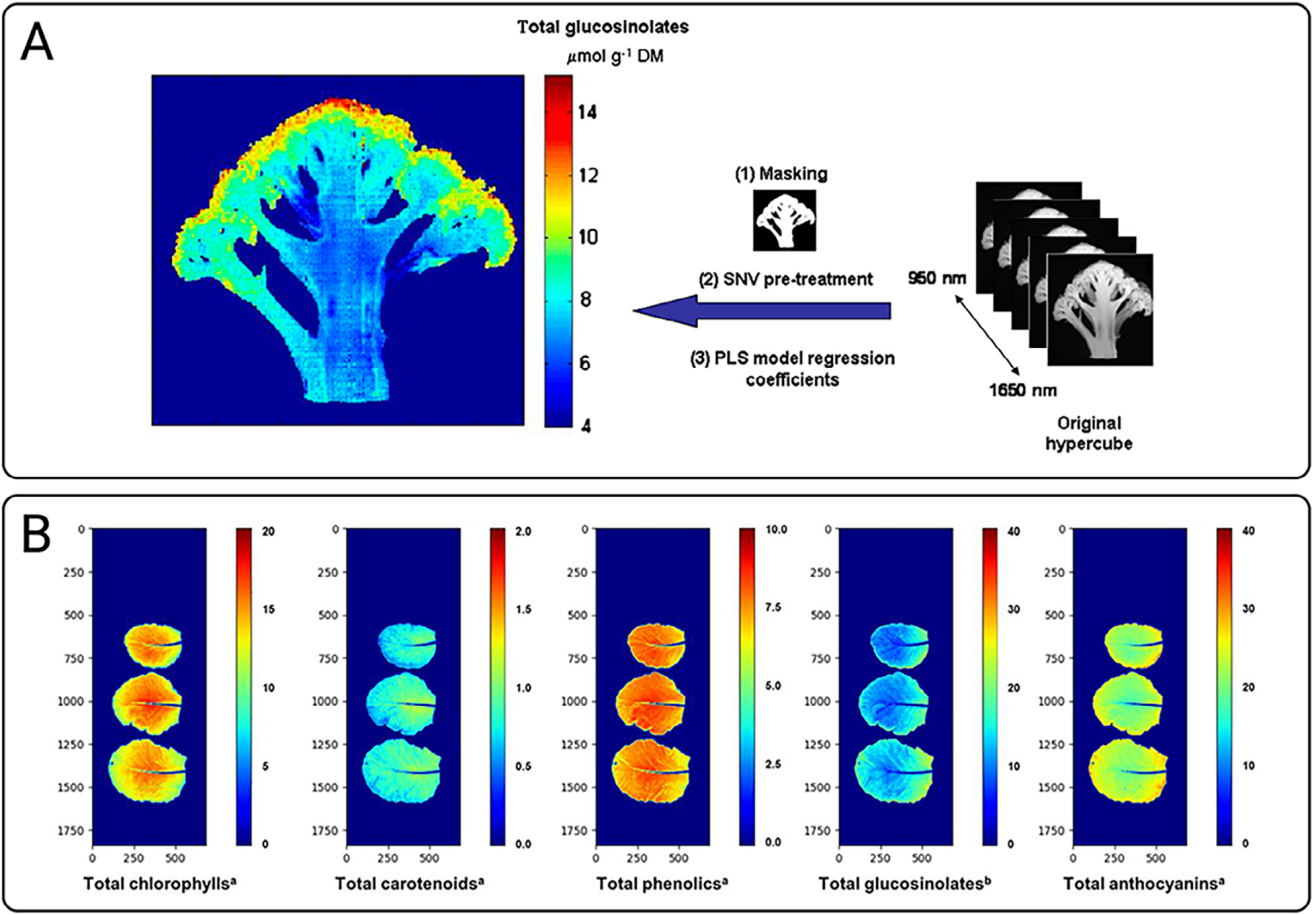
Examples illustrating the use of HSI for quality inspection through the quantification and localization of chemical components. A) A glucosinolate prediction map in a broccoli floret from a PLS model.^[^ ^32^ ^]^ Reproduced with permission from Hernandez‐Hierro et al.^[^ ^32^ ^]^ Copyright 2014, Elsevier B) Distribution maps for different functional components present in *Brassica juncea* leaves as predicted by the PLS model.^[^ ^142^ ^]^ Reproduced under the terms of the CC‐BY Creative Commons Attribution License.^[^ ^142^ ^]^ Copyright 2022 by the authors, published by MDPI.

HSI has been used to inspect the quality of freeze dried grass carp fillets to predict textural attributes (e.g., hardness, guminess, chewiness) for different drying periods.^[^ ^141^ ^]^ Using mean and median spectra for all 381 spectral wavelengths, a quantitative PLSR model was constructed. Rather than constructing separate models for individual textural attributes, one integrated group of wavelengths was selected to build a distributional map. The severe textural alterations induced by lyophilization enhanced the predictive capability of the model. HSI has also been employed to quantitatively localize functional components in food (e.g.,^[^ ^32^, ^142^ ^]^, see Figure 10). For instance, HSI was used to map the presence of glucosinolates, nitrogen and sulfur‐containing bioactive molecules responsible for bitter flavor, in freeze‐dried broccoli florets.^[^ ^32^ ^]^ Two distinct spectral regions were utilized: Vis‐NIR (450–900 nm) and NIR (950‐1650 nm).^[^ ^32^ ^]^ PLS was applied in both cases, with the NIR region yielding superior results (8 terms versus 7 terms).^[^ ^32^ ^]^ These HSI experiments highlighted the importance of spatial mapping, revealing that glucosinolates were predominantly localized in the external parts of the broccoli florets.^[^ ^32^ ^]^ HSI (ranging from 400‐1000 nm) has been employed to detect five representative functional components in *Brassica juncea* leaves (from different cultivation environments) after four days of freeze‐drying.^[^ ^142^ ^]^ The leaf edge and vein were excluded from the analysis due to the lack of functional components present there. The prediction model used a PLS method with 10 preprocessing combinations. This PLS approach achieved high accuracy with minimal data and can be applied relatively easily.

The heterogeneity of freeze‐dried apple slices of different functional components has been characterized.^[^ ^140^ ^]^ The apple slices were analyzed using NIR‐HSI imaging (with a wavelength of 1000–2500 nm and spectral resolution of 12 nm). Randomly extracted spectra were organized in a matrix, and then pretested, smoothed, and preprocessed with SNV to increase signal‐to‐noise ratios. Then PCA was used for Region of interest (ROI) selection and characterization. Leave‐one‐out (LOO)‐PLS regression was used to construct the prediction model and to make prediction maps of the different quality attributes. In another study,^[^ ^145^ ^]^ HSI in the NIR range (400–1000 nm, with a resolution of 2.8 nm) was used to detect quality using key physical and chemical components in lyophilized fermented tea leaves. First, black‐and‐white correction was performed.^[^ ^145^ ^]^ Then centering and zero mean normalization, MSC, smooth, 2 derivative, min‐max normalization and centering methods were used to preprocess the HSI data and to correct spectral differences.^[^ ^145^ ^]^ PLS, SVR, and Random Forest algorithm (RF) models were built on the HSI data and compared, with the RF model reported to have better performance in the analysis on endoplasmic components of tea leaves.

Moreover, HSI has been demonstrated for evaluating the authenticity of lyophilized food products. For example, the authenticity of lyophilized halloumi cheese was assessed using HSI and traditional NIR spectroscopy.^[^ ^144^ ^]^ HSI image acquisition in the 400–1000 nm wavelength range was performed.^[^ ^144^ ^]^ PCA and hierarchical cluster models were applied to the HSI data. The HSI method provided more distinct clusters of two lyophilized halloumi cheese types than the traditional NIR method.

#### 4.2.3 Other Biologically Related Applications

In recent years, HSI and lyophilization have been integrated to advance many other biochemically related scientific and analytical fields. HSI provides detailed, multidimensional information on samples, while lyophilization preserves sample integrity by minimizing substance mobility and preventing microbial growth. Integrating these two techniques can significantly enhance the understanding of biological processes and environmental changes by providing comprehensive information on sample composition, structure, and molecular interactions. For example, 240 points within a lyophilized eyeball sample with an area of 80×240 µ*m* ^2^ were scanned using an excitation laser spot at 0.1 mW.^[^ ^149^ ^]^ The resulting Raman map showed distinct tissue components, demonstrating its capability to evaluate the spatial distribution of biological structures and chemical components in tissue samples.

HSI based on confocal Raman spectroscopy and freeze‐dried stratum corneum have been applied to study dermal drug delivery. A proof‐of‐concept study addressed the challenge of Raman signal attenuation with increasing depth into the skin, essential for precise quantification of drug absorption and risk assessment of chemicals and ingredients.^[^ ^150^ ^]^ Feasibility was demonstrated of using lyophilized human skins combined with confocal Raman spectroscopy for quantitative substance depth profiling. Based on these studies, an artificial skin surrogate and an algorithm to correct the attenuation of Raman intensity depth profiles were developed.

Additionally, the combination of HSI and lyophilization has been extended to understand past bioecosystem dynamics.^[^ ^151^ ^]^ In this scenario, sediment cores from the deepest parts of the lake were lyophilized prior to HSI analysis. Their results highlight the importance of characterizing sediment composition with HSI before assessing chlorophyll‐α concentrations, as the accurate characterization of sediment composition can vary the accuracy of chlorophyll‐α estimates. A summary of the representative lyophilization applications of HSI in food industries and other biologically related fields is presented in Table 2.

## 5 Future Perspectives

Although lyophilization has been widely used in various industries, applications of HSI seem to be most beneficial in pharmaceutical manufacturing, in which the regulations are usually much more stringent, and thus necessitate rigorous monitoring and control. With the growth of biopharmaceuticals, lyophilization is expected to become more prominent in the manufacturing process. The Quality by Design (QbD) concept, as required by regulatory agencies, also applies to lyophilization. HSI offers excellent opportunities for in‐situ monitoring in this context. In this context, HSI has the potential in complementing or replacing currently available technologies, with a focus on two aspects: (1) improving existing lyophilization systems and (2) developing novel lyophilization processes.

Typical lyophilization systems usually consist of tools for measuring product temperature, sublimation flux, and residual moisture. HSI can help improve existing lyophilization processes by providing more information about products that cannot be obtained from standard techniques e.g., homogeneity, solid structure, and stability.^[^ ^2^ ^]^ Besides monitoring and control, additional knowledge from HSI could also be useful for guiding the process/formulation design.^[^ ^121^ ^]^ These benefits make HSI a prominent process analytical technology that plays an important role since the design and development phase to the actual operation, ensuring the process is well designed and the final product quality meets the regulations.

HSI could also play a crucial role in the development of novel lyophilization processes. For example, lyophilization is now primarily conducted in batch‐wise manner, but the future holds promise for the adoption of continuous lyophilization processes.^[^ ^154^, ^155^, ^156^ ^]^ Integrating HSI into continuous lyophilization setups could enable real‐time monitoring of product characteristics to facilitate precise and efficient process control and continuous optimization (e.g., Ref. [2]), ultimately leading to improved efficiency and product quality. This advancement in process analytical technology is more important for continuous operation, where a process is expected to operate all the time with minimal interruption or human intervention, than for batch operation.

Data‐driven modeling methods are the key to gaining deep insights into complex lyophilization processes or lyophilized products from HSI data. Current research shows that the synergy of HSI and data modeling technology is changing the practice of lyophilization analysis and process control. However, the application of technology and methods is still in the initial stage of developments. Due to the large amount of data, high‐order difficulty, and parameter complexity of HSI data, it is still challenging to develop effective and proper HSI data modeling methods. Currently, HSI technology is mainly used in offline and small‐scale lyophilization analysis applications, and HSI data analysis mainly relies on traditional modeling methods. The future trend of lyophilization technology application is from batch lyophilization to continuous lyophilization, which correspondingly requires the development of faster, more efficient, and more automated HSI data modeling methods to meet the requirements of online and real‐time analysis of huge high‐dimensional data streams in continuous lyophilization processes.

Due to its advantage in processing high‐order complex data, tensor modeling is one of the promising solutions for solving the problem of automated real‐time online HSI data analysis in continuous lyophilization applications in the future. The recent research has shown the potential of tensor modeling technology in solving complex HSI data modeling in the context of such as advanced manufacturing processes, real‐time industrial pattern recognition, and automated biomedical image analysis.^[^ ^157^, ^158^, ^159^, ^160^ ^]^ Continued progress in developing efficient, novel, and robust tensor modeling methods will hopefully overcome the limitations of instantaneous analysis of large amounts of high‐dimensional HSI data in continuous lyophilization, making HSI technology suitable in this context. Therefore, future research on HSI data modeling methods in lyophilization scenarios can be to develop more efficient and robust tensor modeling methods to cope with huge computing consumption and complex data flow modeling, such as developing parallel and distributed algorithms for large‐scale tensor computing, extending two‐way model optimization methods to high‐order tensor models, and developing specific tensor models for complex irregular tensor modeling (such as nonlinear high‐order tensors data stream from biochemical manufacturing).

Another noteworthy research direction is the application of deep learning methods. In deep learning, the implicit spectral and spatial feature information in the HSI data in lyophilization applications can be extensively and automatically learned. Deep learning methods have significant advantages in handling large scale data with non‐linearity and missing values. Combining tensor modeling technology with other deep learning tools, such as using the multi‐dimensional feature representation obtained by tensor technology as input learning for deep learning model, will greatly improve the computing efficiency, multi‐way interpretability, model accuracy and generalization ability of deep learning models and enhance the insights gaining from complex lyophilization HSI data stream. Recent studies has demonstrated the significant advantage of combining tensor modeling and deep learning tools in a set of different scenarios.^[^ ^114^, ^161^, ^162^ ^]^ There is no doubt that the development and application of deep learning methods will further change the practice of large‐scale HSI data modeling and the paradigm of HSI technology in lyophilization applications.

Recent advancements in HSI technology have opened up new possibilities for characterizing, analyzing, monitoring, and designing lyophilization processes in large‐scale continuous manufacturing under complex conditions. With the requirements for more sophisticated detection or faster process control in lyophilization applications, the emergence of new HSI technologies will bring new solutions. For example, the newly developed wide‐field MIR‐HSI system achieves ultra‐fast spectral imaging rates and produces high‐definition images at over 10 kHz frame rates based on a megapixel silicon camera that surpass video frame rates.^[^ ^163^ ^]^ 100 channels of spectral images are recorded in 10 ms. Additionally, an integrated HSI technology known as SpectraTrack is expected to overcome the traditional trade‐offs between spatial, temporal, and spectral resolution, further expanding the capabilities of high‐throughput HSI.^[^ ^164^ ^]^ High‐speed NIR‐HSI using a spectral phasor transformation^[^ ^165^ ^]^ and fast nano‐IR HSI using a spatial–spectral network^[^ ^166^ ^]^ provide new technical opportunities for the characterization and analytical control of complex and micro‐scale lyophilization materials. These innovations are poised to enhance the efficient and high‐throughput characterization and monitoring of biomaterials and biopharmaceuticals in continuous lyophilization processes, improving the automation of large‐scale industrial lyophilization operations. They will also potentially strengthen the ability to analyze and control more complex dynamic lyophilization processes and products. However, high‐speed and high‐resolution HSI generates massive high‐dimensional data streams, particularly posing challenges for online lyophilization applications. Extracting valuable insights in real time to guide process analysis and control will require the development of more efficient modeling methods and computer algorithms, including techniques for preprocessing, regression and classification, spectral unmixing, and data fusion. The future application of these innovative HSI technologies in large‐scale complex continuous lyophilization processes will depend to a certain extent on the progress of the data modeling and information extraction methods and algorithms. Advanced data modeling approaches, particularly those leveraging efficient tensor computing and deep learning, are expected to play an important role in the widespread adoption of these high‐performance HSI technologies in lyophilization applications. For many HSI applications, not all of the wavelengths are needed to be able to build a predictive model that is sufficiently accurate for the intended application. For this reason, many commercial HSI sensors only provide data for a limited number of wavelengths, which increases data processing speed. High spectral resolution is most desirable when key spectral features for distinguishing between the individual components in the material are narrow, such as for the Raman spectra of the different polymorphs of mannitol,^[^ ^167^ ^]^ while lower spectral resolution could be more proper for those materials with broad spectral features.

## 6 Conclusion

For lyophilization applications, HSI technology plays an important role in characterizing physical and chemical properties, understanding the state change, monitoring and controlling lyophilization process, assessing quality of lyophilized products, and automating manufacturing process. This article outlines HSI technology for lyophilization, focusing on representative data‐driven modeling strategies including HSI preprocessing, HSI unmixing, HSI regression and prediction, and HSI data fusion, as well as promising applications of HSI technology in lyophilization scenarios. It is evident from this paper that lyophilization HSI is a highly interdisciplinary field that integrates knowledge and technologies from chemometrics, signal processing, statistical analysis, and biochemical engineering to address complex lyophilization‐related problems. Despite the widespread use of HSI technology in lyophilization processes and product analysis, several challenges remain, particularly in developing algorithms for real‐time processing of high‐dimensional and large‐scale HSI data streams and mitigating the impact of the lyophilization environment and process complexity on imaging quality. Future advancements in HSI technology for lyophilization will largely depend on overcoming these challenges. As new lyophilization processes and automation in biochemical manufacturing industries develop, innovations in the field of lyophilization HSI will continue to emerge. This work is a small step, and we hope it provides valuable inspiration for future research in the field of lyophilization HSI and its data‐driven modeling strategies.

## Conflict of Interest

The authors declare no conflict of interest.
